# An Ancient Residue Metabolomics-Based Method to Distinguish Use of Closely Related Plant Species in Ancient Pipes

**DOI:** 10.3389/fmolb.2020.00133

**Published:** 2020-06-26

**Authors:** Korey J. Brownstein, Shannon Tushingham, William J. Damitio, Tung Nguyen, David R. Gang

**Affiliations:** ^1^Institute of Biological Chemistry, Washington State University, Pullman, WA, United States; ^2^Department of Anthropology, Washington State University, Pullman, WA, United States; ^3^Department of Civil and Environmental Engineering, Washington State University, Pullman, WA, United States

**Keywords:** ancient residue metabolomics, archaeology, caffeine, nicotine, pre-contact pipe, psychoactive compound, smooth sumac, tobacco

## Abstract

Residues from ancient artifacts can help identify which plant species were used for their psychoactive properties, providing important information regarding the deep-time co-evolutionary relationship between plants and humans. However, relying on the presence or absence of one or several biomarkers has limited the ability to confidently connect residues to particular plants. We describe a comprehensive metabolomics-based approach that can distinguish closely related species and provide greater confidence in species use determinations. An ~1430-year-old pipe from central Washington State not only contained nicotine, but also had strong evidence for the smoking of *Nicotiana quadrivalvis* and *Rhus glabra*, as opposed to several other species in this pre-contact pipe. Analysis of a post-contact pipe suggested use of different plants, including the introduced trade tobacco, *Nicotiana rustica*. Ancient residue metabolomics provides a new frontier in archaeo-chemistry, with greater precision to investigate the evolution of drug use and similar plant-human co-evolutionary dynamics.

## Introduction

There is an increasing recognition of the deep-time co-evolutionary relationship between humans and certain psychoactive and medicinal plants, yet most of what we know about their past uses is rooted in analogy with practices observed in the present or recent past. Despite the fact that tobacco use is a concern worldwide, with significant consequences to health and society, very little is known about its early adoption and use. Using what is called the biomarker approach, we addressed this question in the Pacific Northwest of North America, where we found nicotine-positive smoke implements predating the contact period (Tushingham et al., 2018). Although we could conclude that deep-time continuity of indigenous smoking existed in a place where tobacco has been depicted as being introduced by early Euro-American traders and explorers and that “the spread of domesticated trade tobacco seems to have overtaken and obscured ancient indigenous tobacco practices” (Tushingham et al., 2018), the approach we previously used, based on biomarkers, could not distinguish between trade tobacco and local native *Nicotiana* species.

Other early forays into ancient psychoactive plant use also followed the biomarker approach, where the presence of individual metabolites is connected to specific plant use or specific application. Beyond smoke plant use and associated biomarkers, such as nicotine, anabasine, arbutin, and cotinine (Eerkens et al., 2012; Rafferty et al., 2012; Tushingham et al., 2013, 2018; Carmody et al., 2018), decoction use has been addressed with biomarker analysis, such as identification of theobromine, theophylline, and caffeine being connected to cacao and yaupon holly trade in the southwestern and southeastern United States (Washburn et al., 2014; Crown et al., 2015). However, the biomarker approach has failed to distinguish between related species, leaving questions about human management, domestication, trade, conveyance, cultivation, and ritual and ceremonial use. Medicinal applications are still largely inaccessible with that approach as well.

At the time of Euro-American contact, indigenous peoples in North America smoked as many as 100 different plant species (Moerman, 1998), but much remains to be known about the pre-contact use of these different species. In the Pacific Northwest of North America, *Arctostaphylos uva-ursi (AUV), Cornus sericea (CSE), Nicotiana attenuata (NAT), Nicotiana quadrivalvis (NQU), Nicotiana rustica (NRU), Nicotiana tabacum (NTA), Rhus glabra (RGL)*, and *Taxus brevifolia (TBR)* were the most common smoke plants used by indigenous peoples (Moerman, 1998). These species are listed in Table 1. Figures S1–S8 (maps adapted from Kartesz, 2015) show the current distribution of these plant species in the United States. Furthermore, while pipes as old as 4,000 to 5,000 years have been excavated in archaeological contexts throughout the region, we know very little about what plants people smoked prior to the introduction of trade tobacco by Euro-American traders and explorers after the 1700s. The four *Nicotiana* species cannot be distinguished from each other using the traditional biomarker approach because this approach largely relies on the detection of nicotine, which is a metabolite common to all of these species. In addition, the biomarker approach, by focusing on positive detection and identification of one or a few specific metabolites, cannot reliably tell if multiple plant species have been used in a particular pipe. A metabolomics-based approach, as described herein, has the potential to distinguish species.

**Table 1:** Compounds shared between the ancient pipes (i.e., PIPE_116 and PIPE_108) and plants smoked in this study.

**Common name**	**Species**	**Abbreviation**	**Family**	**Commonly associated biomarkers**	**Tissue smoked**	**Compounds exclusive to each experimental pipe**	**Compounds common to the pre-contact pipe (PIPE_116) and each experimental pipe**	**Compounds common to the post-contact pipe (PIPE_108) and each experimental pipe**
Bearberry/kinnikinnick	*Arctostaphylos uva-ursi* (L.) Spreng.	*AUV*	Ericaceae	Arbutin	leaves	108	5	1
Red osier dogwood	*Cornus sericea* L.	*CSE*	Cornaceae	*	Bark	65	6	1
Coyote tobacco	*Nicotiana attenuata* Torr. ex S. Watson	*NAT*	Solanaceae	Nicotine	leaves	71	0	0
Indian tobacco	*Nicotiana quadrivalvis* Pursh.	*NQU*	Solanaceae	Nicotine	leaves	152	8	0
Aztec tobacco	*Nicotiana rustica* L.	*NRU*	Solanaceae	Nicotine	leaves	46	3	5
Cultivated tobacco	*Nicotiana tabacum* L.	*NTA*	Solanaceae	Nicotine	leaves	39	2	1
Smooth sumac	*Rhus glabra* L.	*RGL*	Anacardiaceae	*	Autumn leaves	452	33	2
Pacific yew	*Taxus brevifolia* Nutt.	*TBR*	Taxaceae	Paclitaxel	Needles	545	5	0

Commonly associated biomarkers for archaeometric studies were found in KNApSAcK (Afendi et al., 2011). The asterisks (*) indicate that the biomarkers for Cornus sericea (CSE) and Rhus glabra (RGL) could not be determined in KNApSAcK.

The ability to identify ancient plant use to the species-level has a wide range of potential applications. For example, ancient residue metabolomics provides expanded ability to ask anthropologically significant questions about the past use, spread, and conveyance dynamics of *specific plant species* by humans over broad time scales, giving researchers the tools to test assumptions about sites, artifact function, and ritual activity that associate certain plant species with specific artifact types.

We outline here a comprehensive, metabolomics-based method for ancient residue analysis that can differentiate use of specific plants from closely related species. This approach was applied to two archaeological pipes, a “post-contact” pipe (dating to the time following the arrival of Euro-Americans, and their trade goods, which occurs in the Northwest region by the end of the eighteenth century C.E.) and a “pre-contact” pipe, which dates to 1334–1524 calibrated radiocarbon years before present (cal BP) (Pouley, 2001; Damitio, 2018). For the pre-contact pipe, we identified with confidence which among several closely related *Nicotiana* species, as well as additional non-solanaceous species, were smoked in the pipe. Analysis of the post-contact pipe identified the use of different plant species. The developed methods (i.e., experimental smoking of pipes, sequential extraction method, and chemical analysis) described here thus demonstrate the capabilities of metabolomics for archaeometric residue analysis and provide a specific approach that is robust and reliable.

## Materials and Methods

### Plant Growth

Seeds of *Nicotiana attenuata* (USDA Agricultural Research Services [ARS] National Plant Germplasm System; Accession Number: PI 555476), *Nicotiana quadrivalvis* (USDA ARS National Plant Germplasm System; Accession Number: PI 555485), *Nicotiana rustica* (USDA ARS National Plant Germplasm System; Accession Number: PI 555554), and *Nicotiana tabacum* (Strictly Medicinal, Williams, OR, USA) were sown on Sunshine Mix LC1 soil (sphagnum peat moss and perlite; Sun Gro Horticulture Inc., Agawarm, MA, USA). The plants were grown for 60 days with the following greenhouse conditions—average temperatures: 24/17°C (day/night) and under 1000 W metal-halide lights, which are in the bluer/lower wavelengths, to supplement natural daylight. Lights were set for 16/8 h (day/night) and to come on when the outside light intensity fell below 200 μmol·m^−2^·s^−1^. Light intensity readings averaged 350–400 μmol·m^−2^·s^−1^ in the greenhouse during the day. The plants were fertilized twice a week with Peters 20-20-20 (N-P-K; JR Peters Inc., Allentown, PA, USA) supplemented with iron chelate, magnesium sulfate, and trace elements.

*Arctostaphylos uva-ursi, Cornus sericea*, and *Rhus glabra* were collected on the Washington State University, Pullman campus on April 2015, September 2016, and September 2016, respectively. *Taxus brevifolia* was collected in the Iller Creek Conservation Area, WA, USA on October 2016. Vouchers specimens collected by Korey Brownstein were filed in the Marion Ownbey Herbarium, Washington State University, Pullman, WA, USA and can be found by performing a person search in the following database: herbaria.wsu.edu/web/personSearch.aspx. The commonly associated biomarkers of each smoke plant (Table 1) were determined by searching the database, KNApSAcK (Afendi et al., 2011).

### Smoking of Experimental Pipes

After collecting *A. uva-ursi* leaves, *C. sericea* bark, *N. attenuata* leaves, *N. quadrivalvis* leaves, *N. rustica* leaves, *N. tabacum* leaves, *R. glabra* autumn leaves, and *T. brevifolia* needles, they were freeze-dried for 3 days and crushed for experimental smoking. Following experimental conditions modified from Tushingham et al. (2013), the plant tissues were smoked in the Tushingham Ancient Residue Lab (TARL). Commercially distributed clay pipes (Sanctuary Traders, Tinley Park, IL, USA) were used to guarantee uniformity in terms of design, firing temperatures, and clay composition. The dimensions of the experimental pipes were as follows—bowl diameter: 1.5 cm, bowl depth: 2.0 cm, stem length: 9.5 cm, and total length: 11.0 cm. The pipes were inserted into a vice and connected to catheter tip disposable syringes by adjustable rubber tubing. Plant tissues—either leaves, needles, or bark shavings—were lit using commercial butane gas stove lighters. To prevent any harm to research personnel, the experiment was performed in a fume hood.

For the five experimental pipe replicates of each plant species, five full bowls (dried weights ranged between 87.50 and 256.90 mg) were smoked in the same pipe. After smoking was completed, pipes were cut into three segments—bowl, stem (shaft), and tip (mouthpiece)—using a hacksaw. Pipe fragments are more likely to preserve archaeologically rather than complete pipes. Thus, archaeologists are concerned with which areas of artifacts compounds will most likely be present. Pipe studies were designed to test the three main sections (bowl, stem, and tip) to better understand which portions should be selected for analysis. In addition to the three pipe segments, all materials, including syringes, tubing, stirring picks and ashes, were labeled and stored.

### Extraction of Experimental Pipes

The bowl, stem, and tip segments from each pipe were placed into three separate conical tubes, submerged in 2% aqueous tartaric acid (TA) up to the 15.0, 6.0, and 5.5 mL marks, respectively, and then sonicated for 10 min. Each pipe section was then transferred to a new conical tube containing the same amount of acetonitrile:2-propanol:water [3:2:2] (APW) and sonicated for 10 min. Afterwards, each section was transferred to a 2.0 oz (59.15 mL) wide mouth glass jar (Uline Inc., Pleasant Prairie, WI, USA) containing the same amount of methyl *tert*-butyl ether (MTBE) and sonicated once more for 10 min. Small amounts of supernatant (0.60 mL) from the TA and APW extracts were transferred to new 1.5 ml microfuge tubes, frozen at −80°C and freeze-dried for 3 days. In the case of the MTBE extracts, 0.60 mL of supernatant was transferred to a glass vial and dried in the fume hood for 3 days. The extracted experimental pipes were allowed to air-dry on the lab bench. OmniSolv acetonitrile, OmniSolv methyl *tert*-butyl ether, and OmniSolv water (MilliporeSigma, St. Louis, MO, USA) were purchased from VWR International (Radnor, PA, USA). Tartaric acid and Optima 2-proponal were purchased from MilliporeSigma (St. Louis, MO, USA) and Fisher Scientific (Waltham, MA, USA), respectively. All solvents used for extraction were of mass spectrometry grade.

The dried TA and APW extracts were resuspended with 0.60 mL of 0.10% formic acid/water:acetonitrile [1:1] and the dried MTBE extracts were resuspended with 0.60 mL of 0.10% formic acid/acetonitrile. The resuspended samples were vortexed and centrifuged at 10,000 × g for 10 min at 4°C. An amount of 0.50 mL from each sample was placed in a sample vial for analysis. Five non-smoked blank pipes were cut into segments with the hacksaw and extracted as controls following the same extraction methods as the experimental pipes.

### Extraction of Ancient Artifacts

**Figure 1:**
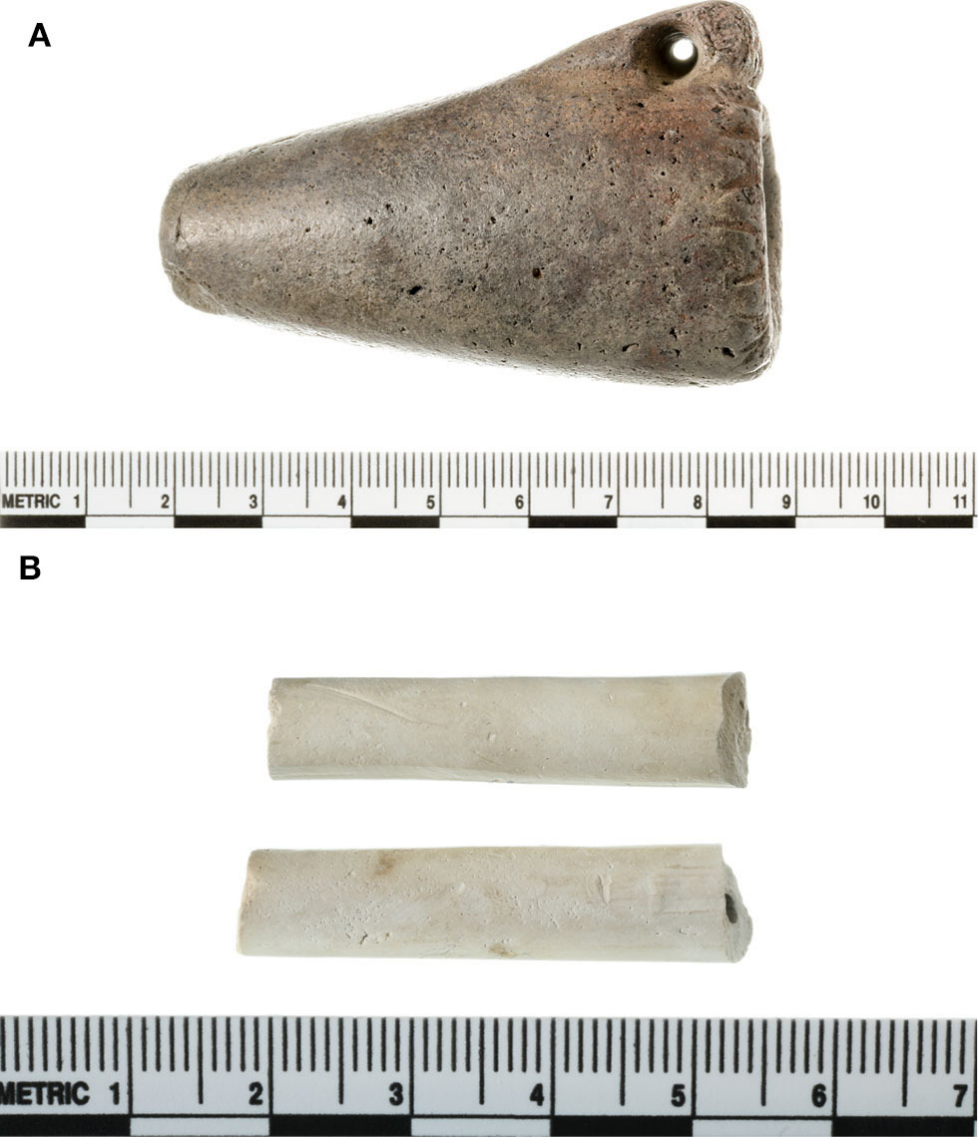
The ancient pipes analyzed in this study. The pre-contact pipe (**A**; Washington State University-Museum of Anthropology Inventory Number: 45GR27.116; PIPE_116) and post-contact pipe (**B**; Washington State University-Museum of Anthropology Inventory Number: 45WW6.108; PIPE_108) are roughly 7.0 and 4.0 cm in length, respectively.

A pre-contact pipe, PIPE_116 (Washington State University-Museum of Anthropology Inventory Number: 45GR27.116; Figure 1A), is associated with a radiocarbon date obtained by Pouley (2001) of 1,520 ± 40 BP [1,334–1,524 cal BP; 2σ calibration (*p* = 1.0) against the IntCal13 curve (Reimer et al., 2013) using CALIB rev. 5.0 (Stuiver et al., 2005)]. A post-contact pipe, PIPE_108 (Washington State University-Museum of Anthropology Inventory Number: 45WW6.108; Figure 1B), was also included in this study. Extraction and analysis of the archaeological artifacts followed a method that was very similar to the experimental pipe analysis, but they were not cut into pieces with a hacksaw. Each artifact was completely submerged in 2% aqueous tartaric acid (TA) and sonicated for 10 min. Each artifact was then transferred to another glass beaker, submerged in acetonitrile:2-propanol:water [3:2:2] (APW), and sonicated for 10 min. Afterwards, each artifact was transferred to a glass beaker, submerged in methyl *tert*-butyl ether (MTBE), and sonicated once more for 10 min. The TA and APW extracts were freeze-dried for 3 days and the MTBE extracts were dried in the fume hood for 3 days. The extracted pre- and post-contact pipes were allowed to air-dry on the lab bench.

The dried TA and APW extracts were resuspended with 0.60 mL of 0.10% formic acid/water:acetonitrile [1:1] and the dried MTBE extracts were resuspended with 0.60 mL of 0.10% formic acid/acetonitrile. The resuspended extracts were vortexed and centrifuged at 10,000 × g for 10 min at 4°C. An amount of 0.50 mL from each sample was placed in a sample vial for analysis.

### LC-MS Analysis of Metabolites

Quality control (QC) standards were prepared by combining 1.0 mL each from the five replicates of three sections into one tube. Each extract (i.e., TA, APW, and MTBE) and experimental pipe (i.e., blank pipes and pipes smoked with *A. uva-ursi, C. sericea, N. attenuata, N. quadrivalvis, N. rustica, N. tabacum, R. glabra*, and *T. brevifolia*) were a separate QC standard. The 15.0 mL QC standards of TA and APW were freeze-dried for 3 days and the 15.0 mL QC standards of MTBE were dried in the fume hood for 3 days. The dried QC standards of TA and APW were resuspended with 5.0 mL of 0.10% formic acid/water:acetonitrile [1:1] and the dried QC standards of MTBE were resuspended with 5.0 mL 0.10% formic acid/acetonitrile. Afterwards, the resuspended QC standards were filtered through a 0.20 μm filter into glass vials. During each sample batch, the 27 QC standards were run with the pre- and post-contact pipe extracts.

The following LC-MS protocol was modified from Brownstein et al. (2017) for ancient residue analysis. LC was performed on a Waters Acquity ultra-performance liquid chromatography (UPLC) system (Waters Corporation, Milford, MA, USA) with photodiode array (PDA) detection ranging between 210 and 400 nm. One microliter of sample was injected through a 2.0 μL sample loop using the full loop injection mode, and the flow rate was 0.32 mL·min^−1^ with 0.10% formic acid/water (A) and 0.10% formic acid/methanol:acetonitrile [2:3] (B) in a slightly concave gradient elution mode. The gradient elution was applied as follows—97% A:3% B to 15% A:85% B from 0.00 to 12.00 min, changed to 3% A:97% B in 0.10 (12.10) min, maintained at 3% A:97% B until 14.00 min, returned to the initial conditions of 97% A:3% B in 0.10 (14.10) min, and then, before the next injection, maintained at 97% A:3% B until 16.00 min. The analysis time was 16.00 min. The autosampler chamber was set to 8°C. Two Waters Acquity UPLC columns: HSS T3, 1.8 μm, 2.1 × 100 mm (T3 column) and BEH C18, 1.7 μm, 2.1 × 50 mm (C18 column) under three column temperatures, 35, 50, and 65°C were compared. LC-MS analysis typically uses C18 (reverse-phase) columns. Nonetheless, we found that the specialized T3 column was better at retaining small molecules, such as nicotine (Figure S9). Our analysis also revealed that the T3 column at 35°C was the ideal condition for analyzing the experimental pipes because this column became unstable at higher temperatures (e.g., degradation above 50°C). Therefore, we used the T3 column at 35°C for the pre- and post-contact pipe extracts.

A Waters Synapt G2-S HDMS Q-TOF with lockspray ionization was operated using electrospray ionization (ESI) in the positive and resolution mode (23,000 FWHM [full width half maximum]). The scan range was from 100 to 1,200 *m/z* with a scan time of 0.3 s. Mass spectral data were collected in profile mode using MS^E^ with a high collision energy ramp (15 to 40 V) for fragmentation. The capillary voltage, sampling cone voltage, and source offset voltage were 3.0 kV, 60 V, and 60 V, respectively. The source temperature was 100°C with a cone gas (nitrogen) flow rate of 50 L·h^−1^. The desolvation temperature was 250°C with a desolvation gas (nitrogen) flow rate of 900 L·h^−1^. The nebulizer gas (nitrogen) flow was 6.0 bar, and the lock mass compound was leucine enkephalin with a reference *m/z* of 556.2771 [M+H]^+^.

Chemical standards of analytical grade anabasine, arbutin, caffeine, cotinine, nicotine, theobromine, and theophylline were purchased from MilliporeSigma (St. Louis, MO, USA). Under our conditions, anabasine (163.125 [M+H]^+^ *m/z*), arbutin (295.084 [M+2Na]^+^ *m/z*), caffeine (195.090 [M+H]^+^ *m/z*), cotinine (177.105 [M+H]^+^ *m/z*), nicotine (163.125 [M+H]^+^ *m/z*), theobromine (181.078 [M+H]^+^ *m/z*), and theophylline (181.078 [M+H]^+^ *m/z*) eluted at 1.48, 1.72, 3.82, 1.45, 1.15, 2.67, and 3.15 min, respectively. OmniSolv acetonitrile, OmniSolv methanol, and OmniSolv water (MilliporeSigma, St. Louis, MO, USA) were purchased from VWR International (Radnor, PA, USA), while Optima formic acid was purchased from Fisher Scientific (Waltham, MA, USA). All solvents used for analysis were of mass spectrometry grade.

### Processing of LC-MS Data

The LC-MS data were processed and aligned in the open-source metabolomics software, MZmine v2.32 (Pluskal et al., 2010), using eight parameters. These parameters (1–8) were listed sequentially in the batch mode queue. In (1) crop filter, the (a) set filters for scans included scan number: 1–1130, retention time: 0.01–12.51 min, MS level: 1, scan definition: not defined, polarity: +, and spectrum type: any; and (b) *m/z* was 100.0000–1200.0000 *m/z*. In (2) mass detection, the (a) set filters for scans included scan number: 1–1130, retention time: 0.01–12.51 min, MS level: 1, scan definition: not defined, polarity: +, and spectrum type: any; and (b) mass detector was centroid with a noise level of 6.0E2. In (3) chromatography builder, the (a) set filters for scans included scan number: 1–1130, retention time: 0.01–12.51 min, MS level: 1, scan definition: not defined, polarity: +, and spectrum type: any; (b) mass list was masses; (c) minimum time span was 0.01 min; (d) minimum height was 5.0E3; and (e) *m/z* tolerance was 0.01 *m/z* or 5.0 ppm. In (4) peak filter, the duration was checked to exclude peaks above the baseline noise ranging from 0.01–12.01 min caused by the mobile phase. In (5) chromatogram deconvolution, the algorithm was baseline cut-off with a minimum peak height of 1.0E4, peak duration range of 0.00–2.00 min, and baseline level of 9.0E3. To determine the thresholds for minimum peak height and noise level, we visually inspected each chromatogram and then performed pilot runs until there was a reduction in the baseline noise. In (6) isotopic peak grouper, the (a) *m/z* tolerance was 0.01 *m/z* or 5.0 ppm; (b) retention time tolerance was 0.05 absolute (min); (c) monotonic shape was checked; (d) maximum charge was 1; and (e) representative isotope was considered the most intense peak. In (7) order peak list, those created by the previous step (i.e., isotopic peak grouper) were alphabetically ordered. In (8) join aligner, the (a) *m/z* tolerance was 0.01 *m/z* or 5.0 ppm; (b) weight for *m/z* was 10; (c) retention time tolerance was 0.05 absolute (min); (d) weight for RT was 10; (e) require same charge state was checked; (f) require same ID was not checked; and (g) compare isotope pattern was not checked.

After application of the join aligner process in MZmine 2, the dataset was exported into Microsoft Excel and mass spectral features shared with the blank pipes were removed from the analysis. The TA, APW, and MTBE extracts from each sample were combined into a single compound list and compounds with no abundance values were removed. The pre- and post-contact residue extract samples were then compared to the QC standards in the open-source web application, Draw Venn Diagram (Van de Peer, 2015), which uses the Perl-Common Gateway Interface (CGI) and can compare up to 30 samples.

The molecular formulas of each metabolite were determined in MZmine 2 using formula prediction in the submenu option, identification (Pluskal et al., 2012). In formula prediction, the parameters were set with charge: 1; ionization type: [M+H]^+^; *m/z* tolerance: 0.0 *m/z* or 3.0 ppm; element C from 0 to 50; element N from 0 to 5; element H from 0 to 100; element O from 0 to 40; and isotope pattern filter checked with isotope *m/z* tolerance: 0.0 *m/z* or 3.0 ppm; minimum absolute intensity: 1.0E4; and minimum score: 95.0%. Predicted molecular formulas were sorted by mass error (ppm).

A second, same dataset from MZmine 2 was exported into Microsoft Excel. After the mass spectral features shared with the blank pipes were removed, the TA, APW, and MTBE extracts from each sample were combined into a single compound list. This dataset was then transposed and imported into vegan v2.5-1 (Oksanen et al., 2018). Ordination plots in the non-metric multidimensional scaling (NMDS) analysis were made using Jaccard and Ward.D methods in vegan. For principal component analysis (PCA), multivariate statistics was performed in MetaboAnalyst 4.0 (Chong et al., 2018) after selecting none (<5,000 features) for data filtering and then Pareto scaling for data scaling. All datasets were submitted to the MetaboLights (Haug et al., 2012) repository (study identifier: MTBLS890).

### Metabolite Derivatization for GC-MS

Supernatants from the MTBE residue extracts and MTBE QC standards (0.60 mL) were transferred to another tube and dried in the fume hood. To prepare the dried MTBE extracts for derivatization, 20.0 mg of methoxyamine hydrochloride (MeOX) was dissolved into 0.50 mL of pyridine and placed in a water bath for 5 min at 60°C. Then 5.0 μL of the MeOX solution was added to each dried MTBE extract and allowed to shake for 90 min at 600 rpm at 30°C. An amount of 45.0 μL *N*-methyl-*N*-(trimethylsilyl)-trifluoroacetamide (MSTFA) was added to stop the reaction and the MTBE extracts were allowed to shake for an additional 30 min at 600 rpm at 37°C. Afterwards, the MTBE extracts were diluted with 0.55 mL of ethyl acetate/0.010% 1,2,4-trimethylbenzene (internal standard). MTBE blanks were prepared the same way as the MTBE residue extracts and MTBE QC standards. An amount of 0.50 mL from each sample was placed in a sample vial for analysis. High-performance liquid chromatography (HPLC) grade J.T. Baker ethyl acetate (Avantor Inc., Radnor, PA, USA) was purchased from Fisher Scientific (Waltham, MA, USA).

### GC-MS Analysis of Metabolites and Processing of GC-MS Data

The GC-MS protocol was adapted with minor modifications from He et al. (2014). GC-MS analysis was performed using a Pegasus 4D MS TOF (LECO Corporation, St. Joseph, MI, USA) equipped with a Gerstel MPS2 autosampler (Gerstel Inc., Linthicum, MD, USA) and an Agilent 7890A GC (Agilent Technologies, Santa Clara, CA, USA). The derivatized products were separated on a 30 m, 0.25 mm i.d., 0.25 μm df Rxi-5Sil column (Restek Corporation, Bellefonte, PA, USA) with an IntegraGuard pre-column using ultrapure helium at a constant flow of 1.0 mL·min^−1^ as the carrier gas. The linear thermal gradient started with a 1.00 min hold at 50°C, followed by a ramp to 330°C at 20°C·min^−1^. The final temperature was held for 5.00 min prior to returning to initial conditions. Mass spectra were collected at 17 spectra·s^−1^ and the injection port was held at 250°C. The scan range was from 35 to 500 *m/z*. An amount of 2.0 μL of sample was injected at the appropriate split ratio of 1:1. The total run time was 20.00 min, which included an initial 5.00 min acquisition delay. Peak identification was conducted using the Fiehn primary metabolite library (Kind et al., 2009). Peak alignment and spectrum comparisons of MTBE residue extracts, MTBE QC standards, and MTBE blanks were carried out using the Statistical Compare feature of the ChromaTOF software (LECO Corporation, St. Joseph, MI, USA). For detection and alignment, the signal-to-noise ratio and retention time difference were 20 and 0 min, respectively. The similarity score for identification was >700. Each chromatogram was also co-analyzed with chemical standards of analytical grade anabasine, arbutin, caffeine, cotinine, nicotine, theobromine, and theophylline, as well as manually reviewed for individual compounds.

## Results

### Ancient Residue Metabolomics Approach

**Figure 2:**
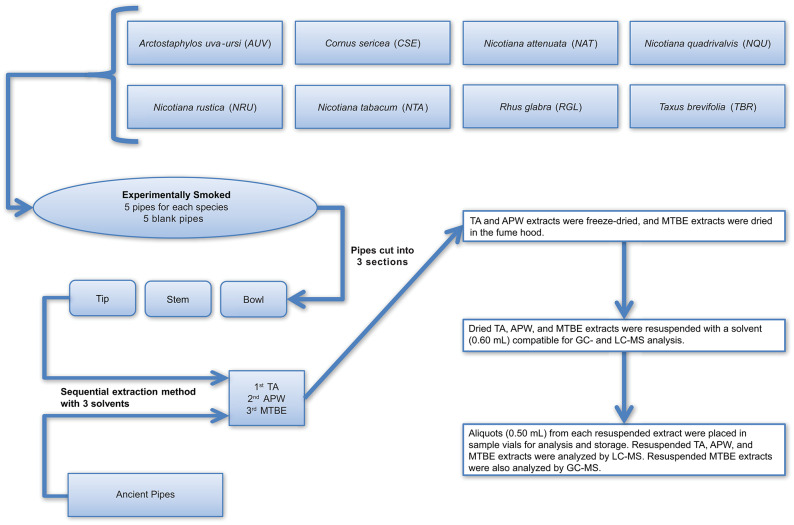
Workflow illustrating how the experimental and ancient pipes were prepared for extraction and analysis. TA, APW, and MTBE correspond to 2% aqueous tartaric acid, acetonitrile:2-propanol:water [3:2:2], and methyl *tert*-butyl ether, respectively.

The method developed for more comprehensive characterization of ancient residues, as outlined in detail in Materials and Methods, begins with the sequential use of 2% aqueous tartaric acid (TA), acetonitrile:2-propanol:water [3:2:2] (APW), and methyl *tert*-butyl ether (MTBE) to extract compounds from the surface of intact artifacts, without the need to physically damage the artifact (such as by scraping, drilling, or abrading). Instead, submersion of the artifact sequentially in each of the solvents with concomitant gentle sonication in a water bath for a period of time dislodges residue components from the artifact surface and dissolves them in the solvent. This does not appear to cause damage to the artifacts. The only observable difference noted by us after analysis of many dozens of artifacts in this manner is that the treated artifacts tend to look “cleaner” (i.e., brighter pigments on pottery sherds, cleaner looking stone pipes, etc.). For some artifacts, such as those that possess externally applied pigments, this cleaning effect may be a concern, and it is possible that removal of residues may expose underlying artifact surfaces to new oxidation in the atmosphere. Therefore, this method may not be suitable for all archaeological samples. However, for stone smoke pipes, which have been exposed to extreme oxidizing conditions in their extended lifetimes, this is not a concern. An overview of the experimental and ancient pipe processing and extraction methods is illustrated in Figure 2.

Despite the caveats associated with the biomarkers approach, this approach is still a useful component of our ancient residue analysis. Beyond biomarkers; however, a multitude of other compounds, mostly unknowns, are detected and compared within a comprehensive set of extractable and analyzable compounds from each residue. Extraction of the artifacts sequentially with three solvents and analysis by multiple mass spectrometry-based platforms ensures broad compound class coverage and allows for detection of a set of compounds (not only one) that help distinguish plant species from one another and provide evidence for multiple plant species use within the same artifact.

An important part of this approach is the inclusion of experimentally smoked pipes. Utilizing these experimental pipes allows for direct comparison of artifacts to known plant species and helps to detect compounds that are indeed present in the putatively smoked plant. Analysis of the experimental pipes revealed in which sections of the pipe analytes were more preserved. Nicotine was detected in all sections of the experimental pipes smoked with *Nicotiana* species. Besides nicotine, we detected arbutin in pipes smoked with *A. uva-ursi* (Figure S10). A number of unknown analytes were also detected in each experimental pipe. Because of this, we prepared quality control (QC) standards as described in Materials and Methods. The QC standards provide a set of compounds present in all sections of an experimental pipe. Furthermore, including the QC standards with the ancient pipe extracts in the GC- and LC-MS sample lists can improve the robustness of the data analysis and reduces chromatogram misalignments in software. The software, MZmine 2, also allows for the specification of ppm and retention time errors, thereby further reducing the likelihood of chromatogram misalignments.

This metabolomics-based approach provides optimal results for extracting various compounds from both potential source plants and archaeological artifacts, both experimental and collected. The TA solvent system was included in the sequential extraction method because it had previously been shown to effectively extract alkaloids (Ma et al., 2005). APW is effective in extracting a large fraction of polar to medium non-polar metabolites, such as flavonoids and other phenolics (Lee and Fiehn, 2008), while MTBE has been used to extract non-polar metabolites (Gang et al., 2001; Jiang et al., 2005; Xie et al., 2009).

**Figure 3:**
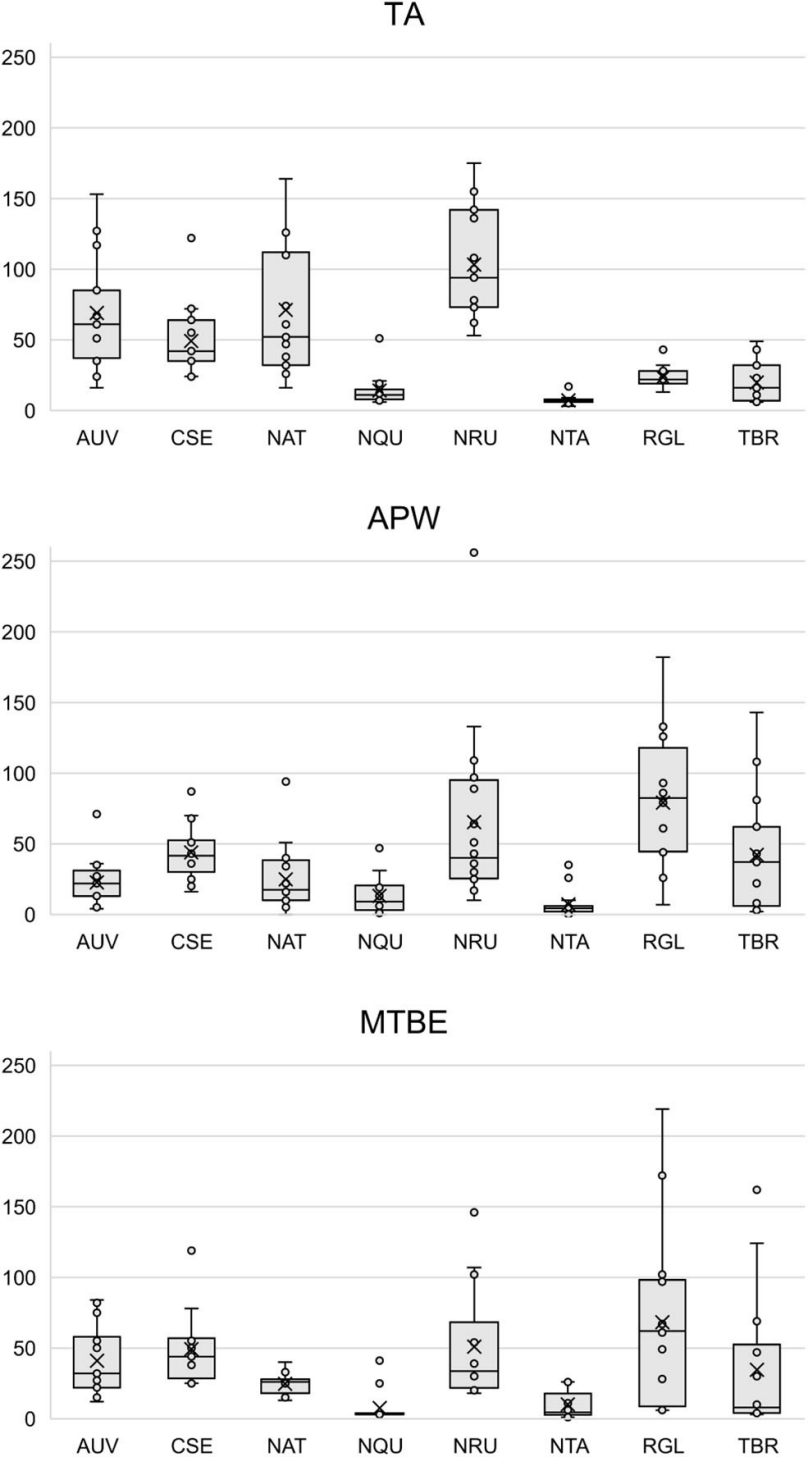
Box plots of the number of mass spectral features detected in each solvent (i.e., TA, APW, and MTBE) by species. Each point represents a sample. The × and horizontal line in each box plot indicate the mean and median, respectively. TA, 2% aqueous tartaric acid [TA]; APW, acetonitrile:2-propanol:water [3:2:2]; MTBE, methyl *tert*-butyl ether; *AUV, A. uva-ursi*; *CSE, C. sericea*; *NAT, N. attenuata*; *NQU, N. quadrivalvis*; *NRU, N. rustica*; *NTA, N. tabacum*; *RGL, R. glabra*; and *TBR, T. brevifolia*.

**Figure 4:**
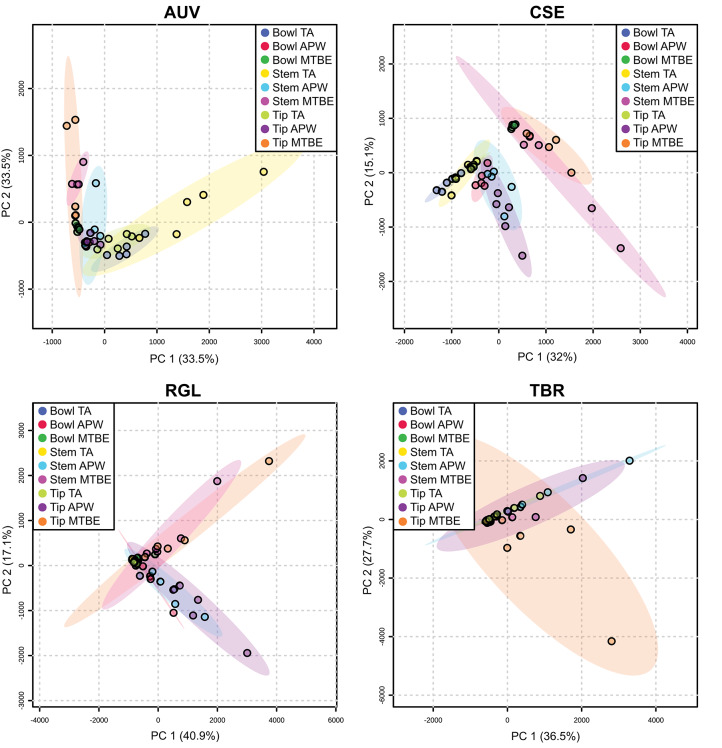
Principal component analysis (PCA) of experimental pipes by section (i.e., bowl, stem, and tip) smoked with *A. uva-ursi* (*AUV*), *C. sericea* (*CSE*), *R. glabra* (*RGL*), and *T. brevifolia* (*TBR*). TA, APW, and MTBE correspond to 2% aqueous tartaric acid, acetonitrile:2-propanol:water [3:2:2], and methyl *tert*-butyl ether, respectively. Each point within the 95% confidence regions represents five different pipe replicates (*n* = 5).

**Figure 5:**
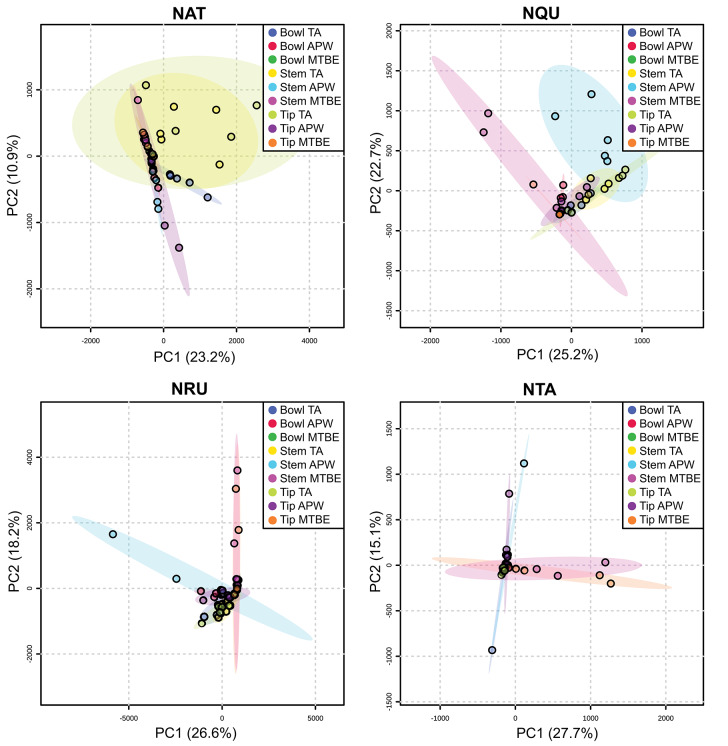
Principal component analysis (PCA) of experimental pipes by section (i.e., bowl, stem, and tip) smoked with *N. attenuata* (*NAT*), *N. quadrivalvis* (*NQU*), *N. rustica* (*NRU*), and *N. tabacum* (*NTA*). TA, APW, and MTBE correspond to 2% aqueous tartaric acid, acetonitrile:2-propanol:water [3:2:2], and methyl *tert*-butyl ether, respectively. Each point within the 95% confidence regions represents five different pipe replicates (*n* = 5).

**Figure 6:**
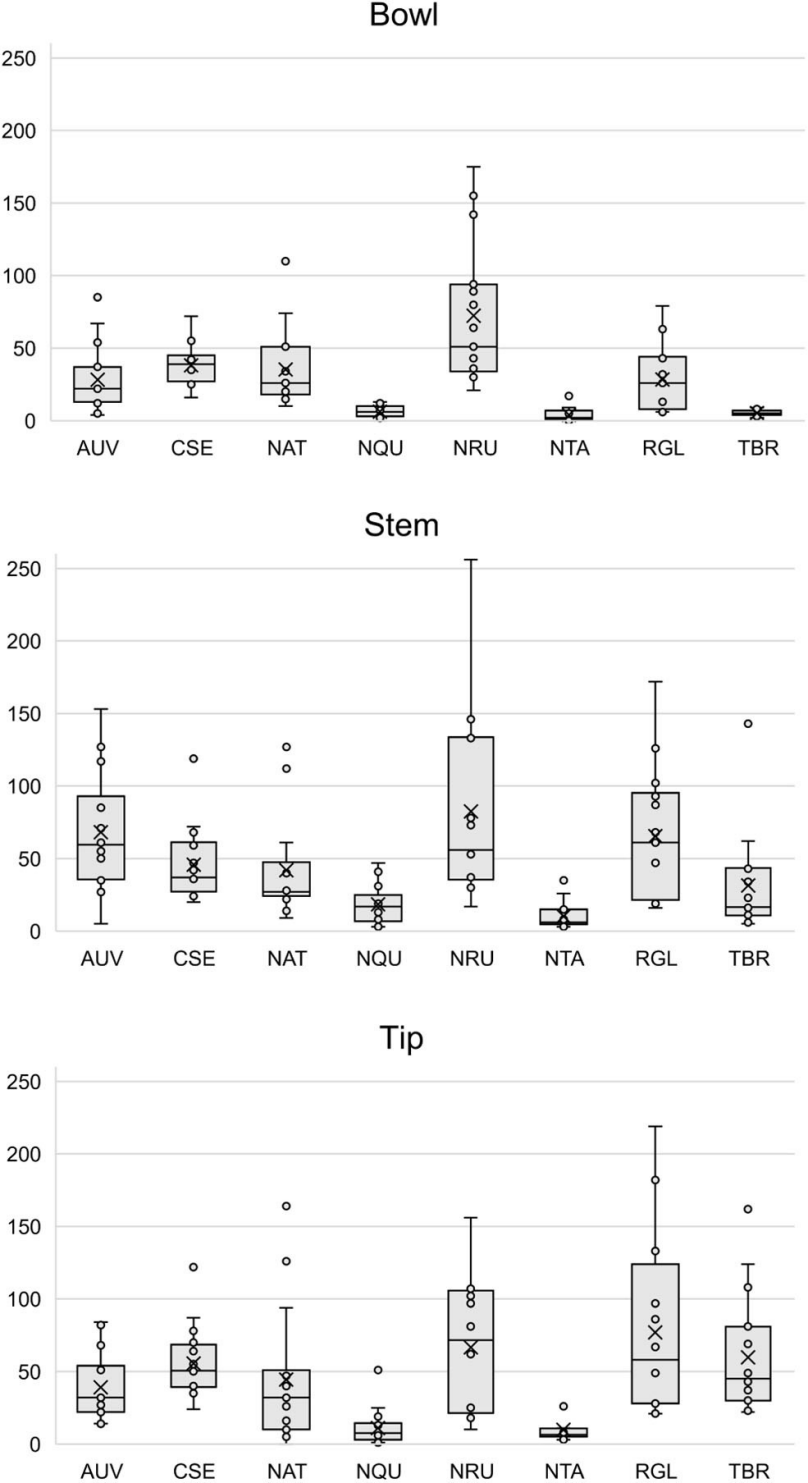
Box plots of the number of mass spectral features detected in each pipe section (i.e., bowl, stem, and tip) by species. Each point represents a sample. The × and horizontal line in each box plot indicate the mean and median, respectively. *AUV, A. uva-ursi*; *CSE, C. sericea*; *NAT, N. attenuata*; *NQU, N. quadrivalvis*; *NRU, N. rustica*; *NTA, N. tabacum*; *RGL, R. glabra*; and *TBR, T. brevifolia*.

Figures S11–S34 show that each solvent in this three-part extraction procedure extracts a range of mass spectral features in the experimental pipes. As shown in the chromatograms (Figures S29–S34), Data Sheet S1 and Figure 3, the APW and MTBE extracts of experimental pipes smoked with *R. glabra* and *T. brevifolia* had more mass spectral features compared to their TA extracts. Figures 4, 5 demonstrate that the sequential extraction method extracted different compounds classes from *A. uva-ursi, C. sericea*, and *N. rustica* experimental pipes, with the APW extracts joining the TA and MTBE confidence regions. Differences in detectable analytes across the experimental pipe sections (i.e., bowl, stem, and tip) can also be observed (Figures S11–S34). To investigate these differences, we counted the number of mass spectral features. The number of analytes was lowest in the bowl for all of the species, with the exception of the experimental pipes smoked with *N. attenuata, N. quadrivalvis*, and *N. tabacum* (Data Sheet S1 and Figure 6). According to Figures 4, 5, the pipe sections did not cluster. In a pilot study, the methanol:chloroform:water [5:2:2] (MCW) solvent system described by Tushingham et al. (2013) was as effective as APW with respect to the number of detectable peaks. However, using a methanol/chloroform solvent system to extract compounds to be analyzed in metabolomics investigations is a concern. That solvent system produces chlorinated waste and can introduce, during the extraction process, methylated compounds as artifacts (Reber and Kerr, 2012).

The chemical standards of anabasine, arbutin, caffeine, cotinine, nicotine, theobromine, and theophylline (biomarkers typically employed in smoke plant or decoction studies) were readily separated and detected using the LC-MS method employed in this approach (Figure S35). GC-MS has been used for biomarker analysis of some of these metabolites in the past. However, distinguishing theobromine from the GC-MS baseline noise (Figure S36) proved more difficult compared to LC-MS. The concentration of theobromine (555 μM) was the same for GC- and LC-MS analysis, but LC-MS was more universal in its detection capabilities, with caffeine, theobromine, and theophylline readily separated with high signal to noise. As shown in Figure S35, anabasine and nicotine have the same *m/z*. However, these biomarkers can be distinguished by their retention times and pseudo MS/MS (MS^E^) spectra (Figures S35B,D). This was also true for theobromine and theophylline (Figures S35F,G).

### Analysis of Pre-contact and Post-contact Pipes

**Figure 7:**
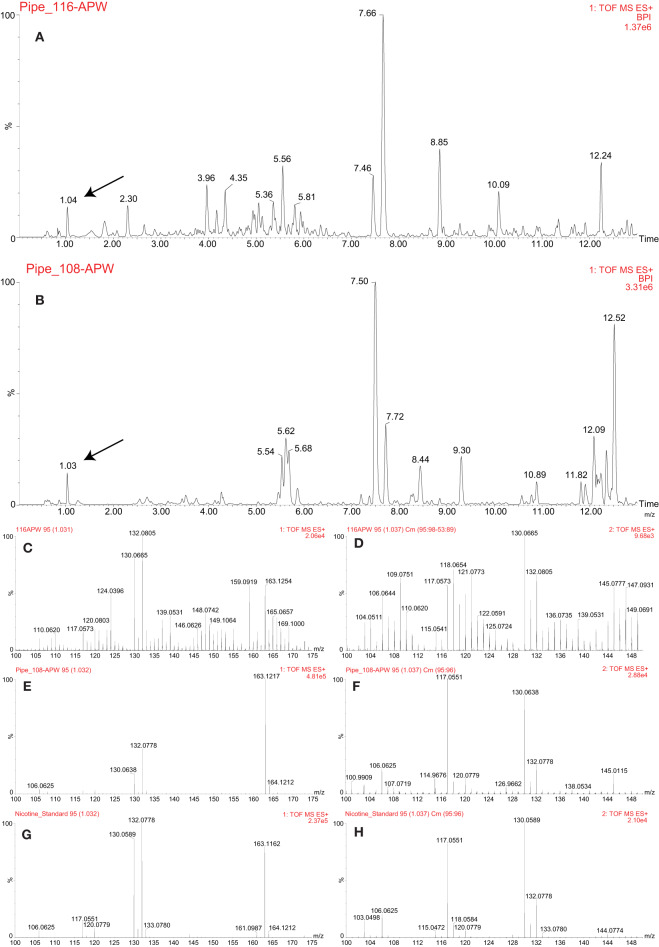
LC-MS analysis of the pre-contact (PIPE_116) and post-contact (PIPE_108) pipes. Base peak intensity (BPI) chromatograms of acetonitrile:2-propanol:water [3:2:2] (APW) extracts from the pre-contact **(A)** and post-contact **(B)** pipes. The black arrows indicate the nicotine peak. MS **(C,G)** and MS^E^ **(D,H)** spectra comparing the nicotine standard **(G,H)** to the peak identified as nicotine in the pre-contact pipe **(C,D)**. MS **(E,G)** and MS^E^ **(F,H)** spectra comparing the nicotine standard **(G,H)** to the peak identified as nicotine in the post-contact pipe **(E,F)**.

The approach outlined above was used to analyze a pre-contact pipe (Figure 1A) and a post-contact pipe (Figure 1B), both from the Pacific Northwest, in efforts to identify which plants were smoked in each and thereby validate and demonstrate utility of this metabolomics-based approach. Numerous mass spectral features were extracted and detected from each pipe, as shown in Figures 7A,B, where the base peak intensity (BPI) chromatograms of the APW extracts for each pipe are displayed and some mass spectra are provided to demonstrate identification of specific metabolites. Further investigation of the peak at 1.04 min (Figure 7A) and 1.03 min (Figure 7B) revealed that nicotine was present in each pipe, suggesting (based on the old single biomarker approach) that a tobacco species had likely been smoked in each pipe. The presence of nicotine in each pipe was confirmed by comparison of MS and MS/MS (MS^E^) spectra to the nicotine standard (Figures 7C–H). Under our conditions, no other biomarkers were identified in the ancient pipes.

**Figure 8:**
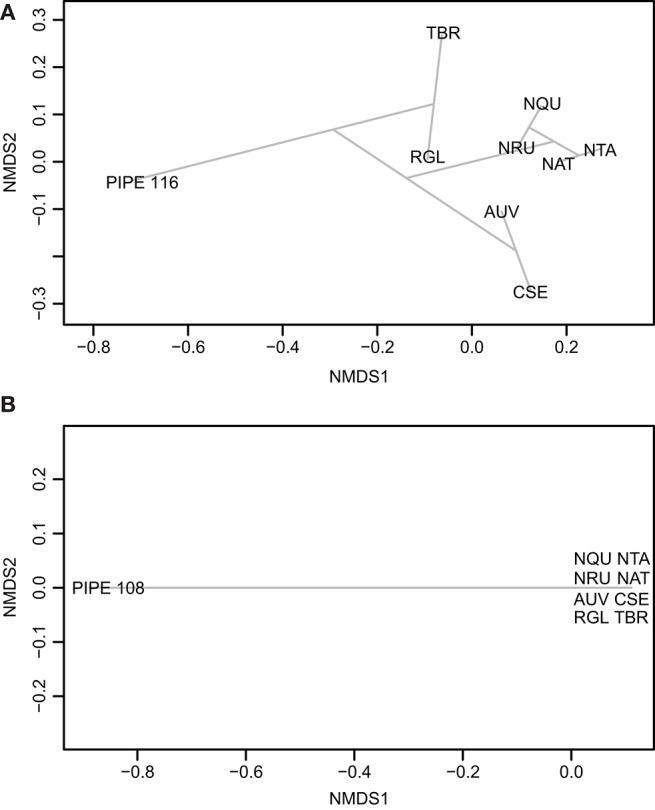
Ordination plots comparing the ancient pipes to the experimental pipes smoked with the plants listed in Table 1. The ordination plots of the pre-contact pipe (**A**; PIPE_116) and post-contact pipe (**B**; PIPE_108) were made using Jaccard and Ward.D methods in vegan. *AUV, A. uva-ursi*; *CSE, C. sericea*; *NAT, N. attenuata*; *NQU, N. quadrivalvis*; *NRU, N. rustica*; *NTA, N. tabacum*; *RGL, R. glabra*; and *TBR, T. brevifolia*.

The full LC-MS chromatograms of the pre- and post-contact pipes were then processed in MZmine 2 to generate metabolomics datasets. These data included the biomarker, nicotine, as well as a large number of non-identified, but reproducibly detected mass spectral features that are thereby useful in non-targeted metabolomics. This comprehensive dataset was analyzed using the program Draw Venn Diagram (Van de Peer, 2015). As shown in Table 1, the Venn diagram analysis revealed that the pre-contact pipe had compounds in common with experimental pipes smoked with the plants, *A. uva-ursi, C. sericea, N. quadrivalvis, N. rustica, N. tabacum, R. glabra*, and *T. brevifolia*. The experimental pipe that had been used to smoke *R. glabra* shared the most compounds with the pre-contact pipe. Thus, in addition to smoking of a *Nicotiana* species, the pre-contact pipe appeared to have been used to smoke other plants, such as *R. glabra*. The post-contact pipe contained compounds indicative of *A. uva-ursi, C. sericea, N. rustica, N. tabacum*, and *R. glabra* smoking (Table 1). Analysis of the data using non-metric multidimensional scaling (NMDS), as implemented in vegan, indicated, based on the separation of the pre- and post-contact pipes from the experimental pipes, that the ancient pipes shared a subset of compounds with the experimental pipes (Figure 8). Under our conditions, a total of 1,241 and 680 mass spectral features were detected in the pre- and post-contact pipe extracts, respectively. The predicted molecular formulas of the subset of compounds common to each experimental and ancient pipe are shown in Table S1. Environmental contaminants, compound degradation, and differences between modern and ancient plant varieties may have contributed to the separation of the ancient pipes from the experimental pipes.

## Discussion

Researchers in archaeometric studies have focused their attention on alkaloids (such as caffeine and nicotine) because they possess psychoactive properties. However, non-alkaloids, including tetrahydrocannabinol (THC) and the salvinorins, possess psychoactive properties as well. Successful detection of not only alkaloids, but also non-alkaloids, depends on using a universal analytical instrument. With GC-MS, Hairfield and Hairfield (2002) could not detect nicotine in ancient pipes from Chile. Echeverría et al. (2014) revisited the analysis performed by Hairfield and Hairfield (2002) and found that their GC-MS analysis had been operated in a less sensitive mode. Sensitivity issues are not uncommon for GC-MS. Alder et al. (2006) found that after analyzing 500 high priority pesticides, including nicotine, LC-MS detected a broader range of compounds and was more sensitive than GC-MS. A study by Perez et al. (2016) also showed that LC-MS performed equivalent to or better than GC-MS at detecting benzodiazepines, a class of psychoactive drugs. Additionally, similar to Perez et al. (2016), we found that LC-MS required less sample preparation and instrument time.

It could be envisioned that Native Americans in a particular region used plants only found in that region. However, this is an inaccurate assumption because Native American communities interacted widely with one another within and between ecological regions, including the trade of tobacco seeds and materials (Harshberger, 1896; Blankinship, 1905; Setchell, 1921; Turner and Taylor, 1972; Haberman, 1984). Furthermore, anabasine, arbutin, caffeine, cotinine, nicotine, theobromine, and theophylline are present in many species at various concentrations throughout the plant kingdom (Afendi et al., 2011). Thus, these biomarkers should not be relied on as specifiers of plant use in a pipe at the species-level, perhaps not even at the genus or higher levels.

The LC-MS/MS (MS^E^) analysis presented herein clearly identified nicotine in the pre- and post-contact pipes (Figures 7C–H), suggesting tobacco use. However, presence of this metabolite was insufficient evidence to discriminate which *Nicotiana* species had been smoked. Processing the data in MZmine 2 and subsequent Venn diagram analysis revealed that *R. glabra* shared the most detected compounds with the pre-contact pipe, indicating that this species was likely smoked in this pipe (Table 1). The pre-contact pipe was excavated from a site in central Washington State (Daugherty, 1952; Damitio, 2018), which overlaps with the current distribution of *R. glabra* (Figure S7). The tobacco species most associated with the pre-contact pipe was *N. quadrivalvis*, which had eight shared compounds (Table 1). A few of these compounds are predicted to be nitrogen-containing molecules (Table S1). This plant species is not currently present in Washington State (Figure S4); however, researchers have reported that tribes of the Northwest widely cultivated *N. quadrivalvis* for smoking (Harshberger, 1896; Blankinship, 1905; Setchell, 1921; Turner and Taylor, 1972; Haberman, 1984). The range of this species may have once extended into Washington State (Turner and Taylor, 1972; Tushingham et al., 2018). The residue extracted from the pre-contact pipe may have been from a mixture of *N. quadrivalvis, R. glabra*, and other plant species, either smoked sequentially or together. According to the ethnobotanical literature, *R. glabra* leaves were commonly mixed with tobacco for its medicinal qualities and to improve the flavor of smoke (Kroeber, 1941).

The ordination plots in Figure 8 illustrate the complexities of the pre- and post-contact pipes. The distance of these ancient pipes from the experimental pipes indicate significant differences between their compound compositions. This distance may have arisen from environmental contaminants, compound degradation, and differences between modern and ancient plant varieties. As expected, the experimental pipes smoked with each *Nicotiana* species clustered in the ordination plots (Figure 8). A Venn diagram analysis of the post-contact pipe detected five compounds shared with *N. rustica*, but zero shared with *N. quadrivalvis* (Table 1). The current distribution of *N. rustica* is in the eastern United States (Figure S5). This species, initially domesticated in South America, likely was introduced to the eastern United States through pre-contact trade routes. By the time of European contact, *N. rustica* was a widely used smoke plant in native farming communities throughout eastern North America (Rafferty et al., 2012). Domesticated South American tobaccos reached the Northwest much later; they were a major trade commodity brought to the area by Euro-American traders and explorers beginning in the 1700s. After this time, the use of indigenous tobaccos (such as *N. quadrivalvis*) was abandoned or became less favorable (Turner and Taylor, 1972; Tushingham et al., 2018). Our results reflect these historic events, indicating that *N. quadrivalvis* had been smoked in the pre-contact pipe, but a likely change in preference for stronger introduced trade tobaccos led to the use of *N. rustica* in the post-contact pipe. The change in local preference toward *N. rustica* can likely be explained by its greater nicotine content—and therefore more potent addictiveness—relative to *N. quadrivalvis* (Sisson and Severson, 1990; Winter, 2000a, p. 317).

Our developed methods are a significant breakthrough for ancient residue analysis. As demonstrated, a broader suite of compounds can be extracted (Figures 4, 5) using the sequential extraction method and LC-MS analysis, respectively. The results presented indicate that metabolomics can help identify more plant taxa and, in some cases, discriminate plant use in ancient artifacts to the species-level. Until now, the use of specific smoking plants in the Northwest in the past has been only speculated about. Our results provide not only the first association of a non-tobacco plant (*R. glabra*) in an archaeological pipe, but also reflect an evolving use of tobacco, e.g., from an endemic species (*N. quadrivalvis*) to a domesticated trade species (*N. rustica*). Further, though it is a commonly held view that trade tobacco (specifically, *N. tabacum*) overtook pre-contact endemic smoke plants after the time of Euro-American contact (Goodspeed, 1954, p. 9, 353; Turner and Taylor, 1972; Winter, 2000b, p. 27; Tushingham et al., 2018), our results run contrary to this expectation, suggesting that *N. rustica* was associated with the post-contact pipe. Such findings demonstrate metabolomics to be a new frontier in archaeometric analysis that can offer an unprecedented window into the evolving use and conveyance of specific psychoactive plants by ancient humans. Therefore, this approach can be used to address numerous potential questions in archaeo-chemistry and beyond.

## Data Availability Statement

All datasets generated for this study are included in the article/Supplementary Material.

## Author Contributions

KB, ST, and DG designed the experiments. KB performed the chromatography experiments and data analyses. WD extracted the ancient pipes. TN modified the vegan script for the datasets. KB wrote the paper with input from ST, WD, and DG. All authors approved of the final version of the paper.

## Conflict of Interest

The authors declare that the research was conducted in the absence of any commercial or financial relationships that could be construed as a potential conflict of interest.
