# Supplementary material for: An Ancient Residue Metabolomics-Based Method to Distinguish Use of Closely Related Plant Species in Ancient Pipes
Source: Front Mol Biosci. 2020 Jun 26;7:133. doi: 10.3389/fmolb.2020.00133 (PMC7332879; doi:10.3389/fmolb.2020.00133)
Supplement: Supplementary file 1 [file Image_1.PDF]

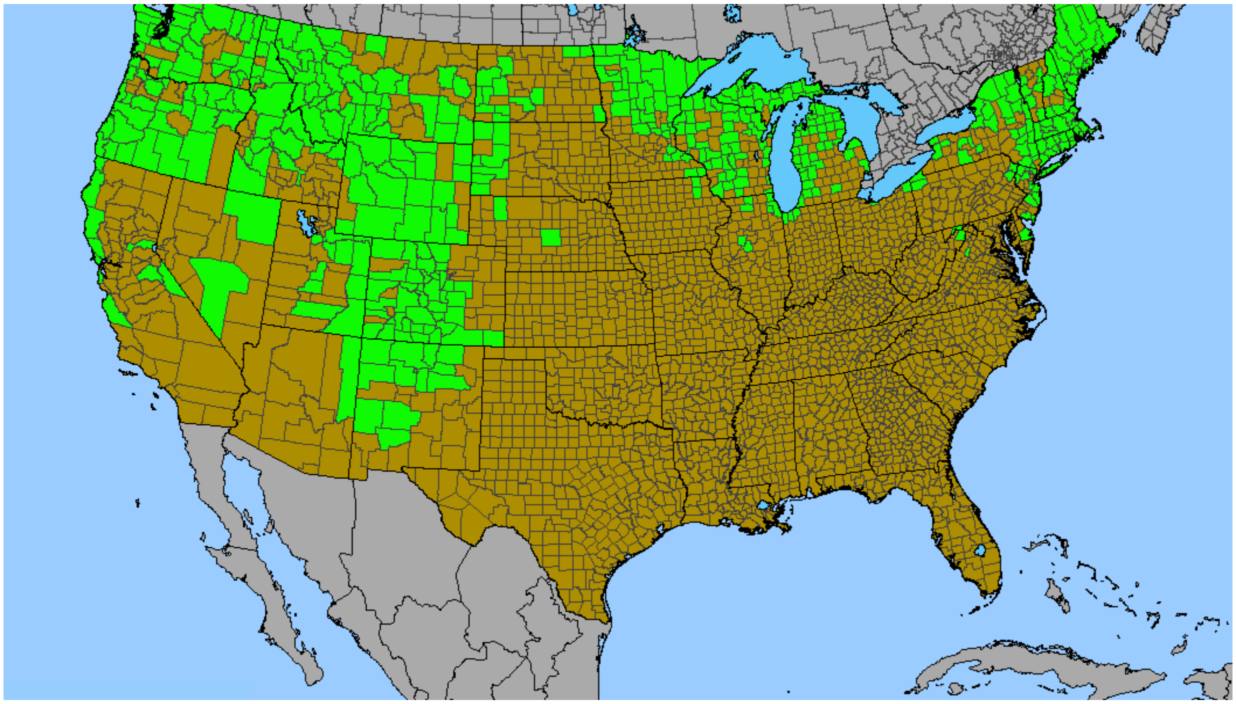

Figure S1. Map adapted from Kartesz (2015) showing the county distribution of *Arctostaphylos uva-ursi* (*AUV*). Green indicates that the species is present and native. Gray and brown represent no data and that the species is not present, respectively.

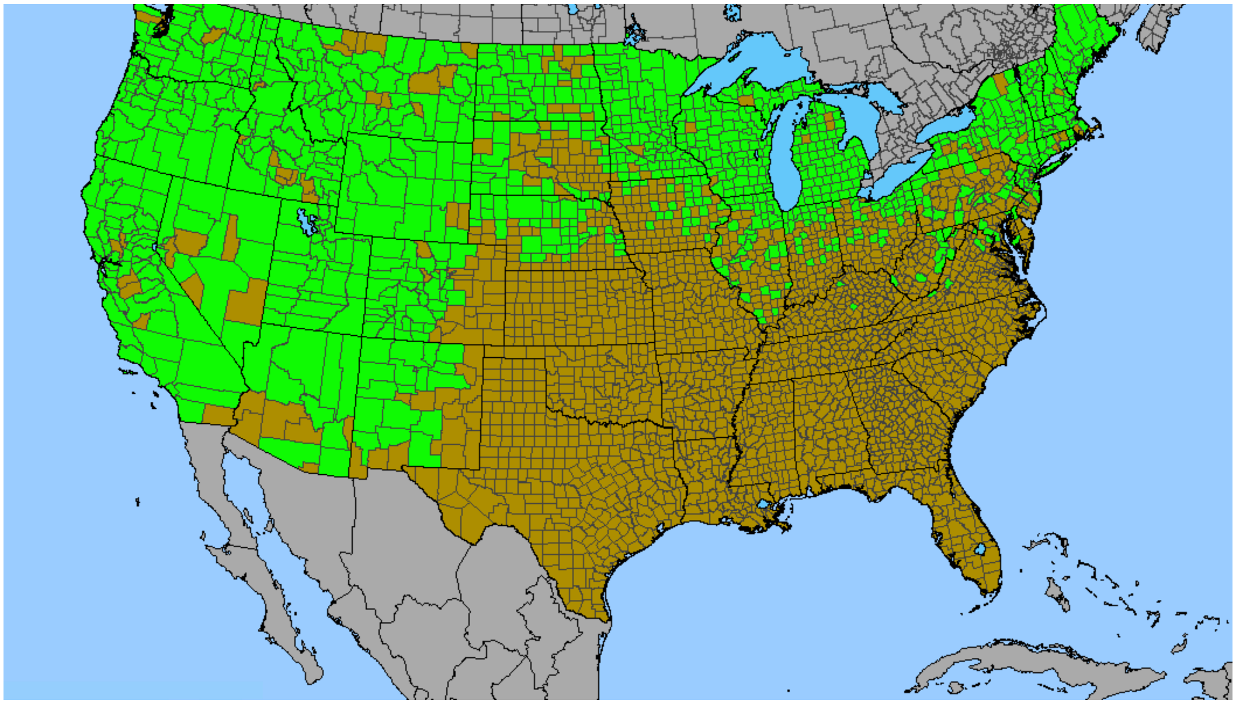

Figure S2. Map adapted from Kartesz (2015) showing the county distribution of *Cornus sericea* (*CSE*). Green indicates that the species is present and native. Gray and brown represent no data and that the species is not present, respectively.

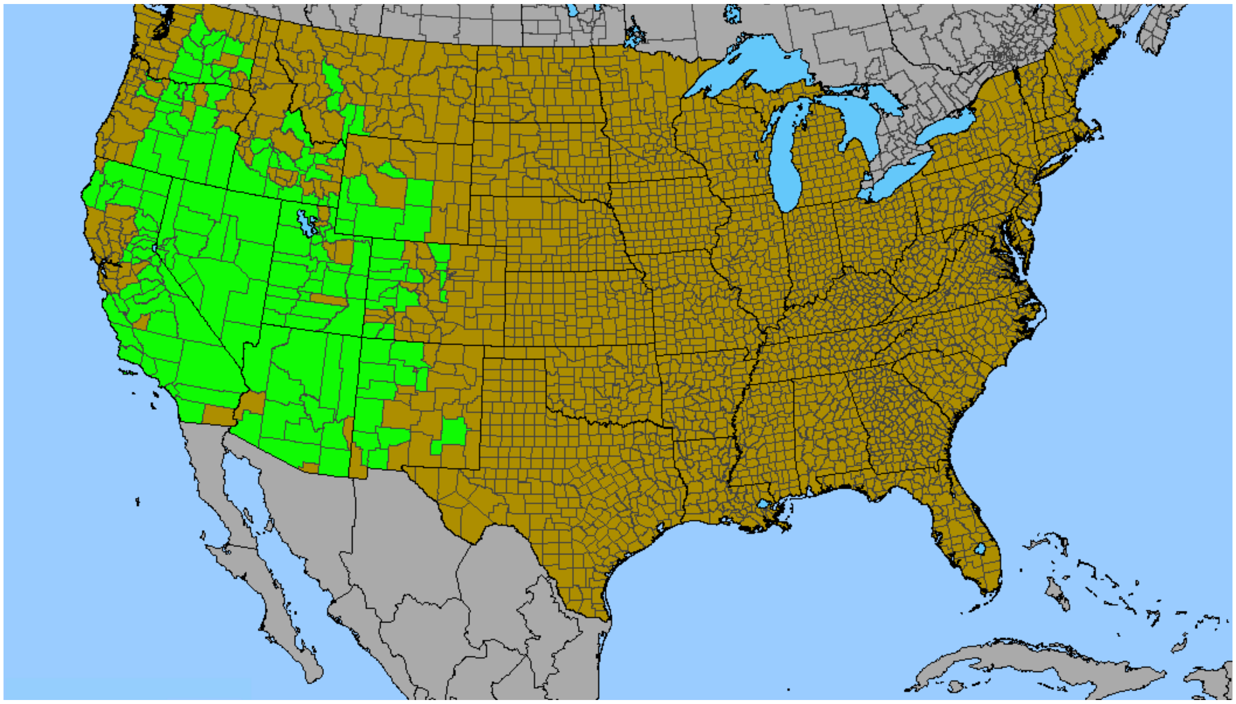

Figure S3. Map adapted from Kartesz (2015) showing the county distribution of *Nicotiana attenuata* (*NAT*). Green indicates that the species is present and native. Gray and brown represent no data and that the species is not present, respectively.

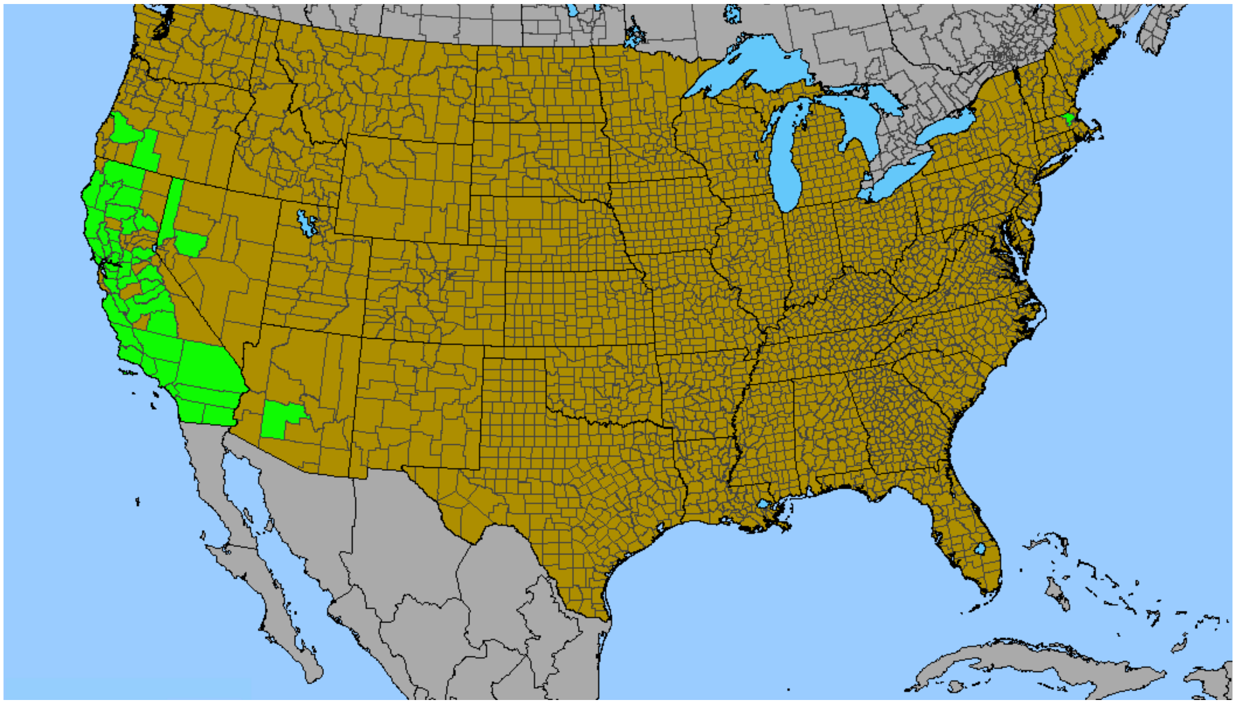

Figure S4. Map adapted from Kartesz (2015) showing the county distribution of *Nicotiana quadrivalvis* (*NQU*). Green indicates that the species is present and native. Gray and brown represent no data and that the species is not present, respectively.

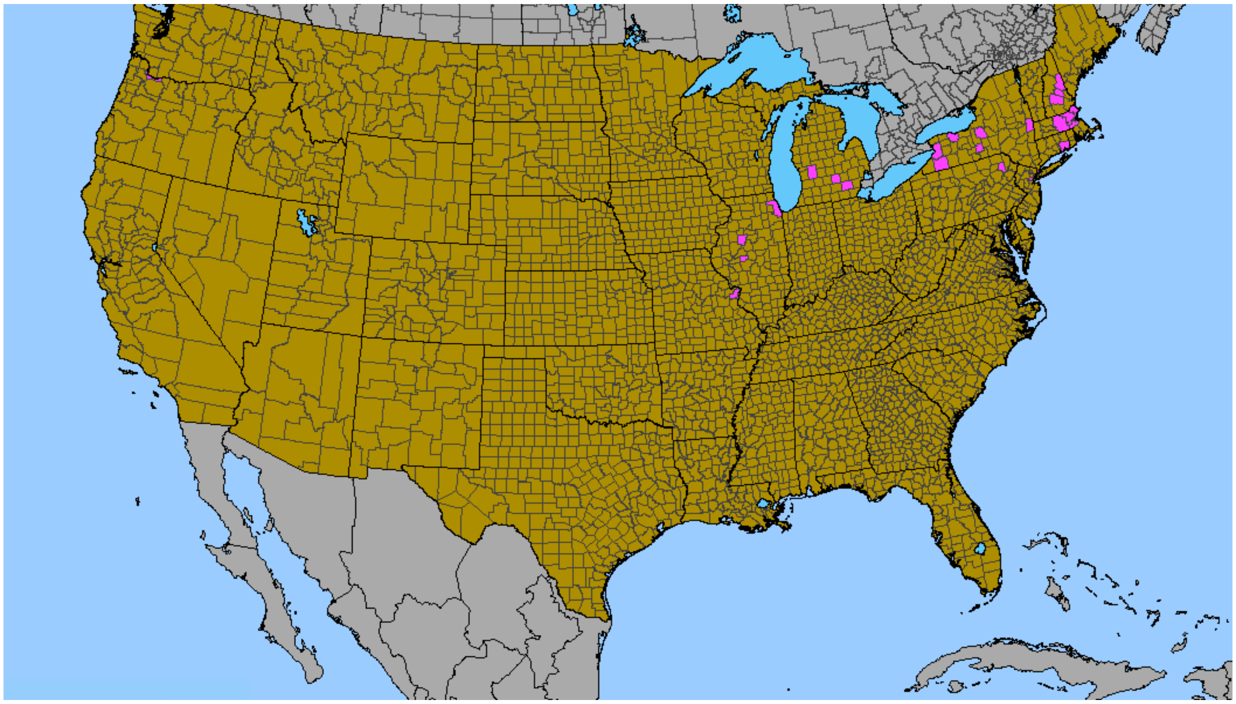

Figure S5. Map adapted from Kartesz (2015) showing the county distribution of *Nicotiana rustica* (*NRU*). Magenta indicates that the species is present and non-native. Gray and brown represent no data and that the species is not present, respectively.

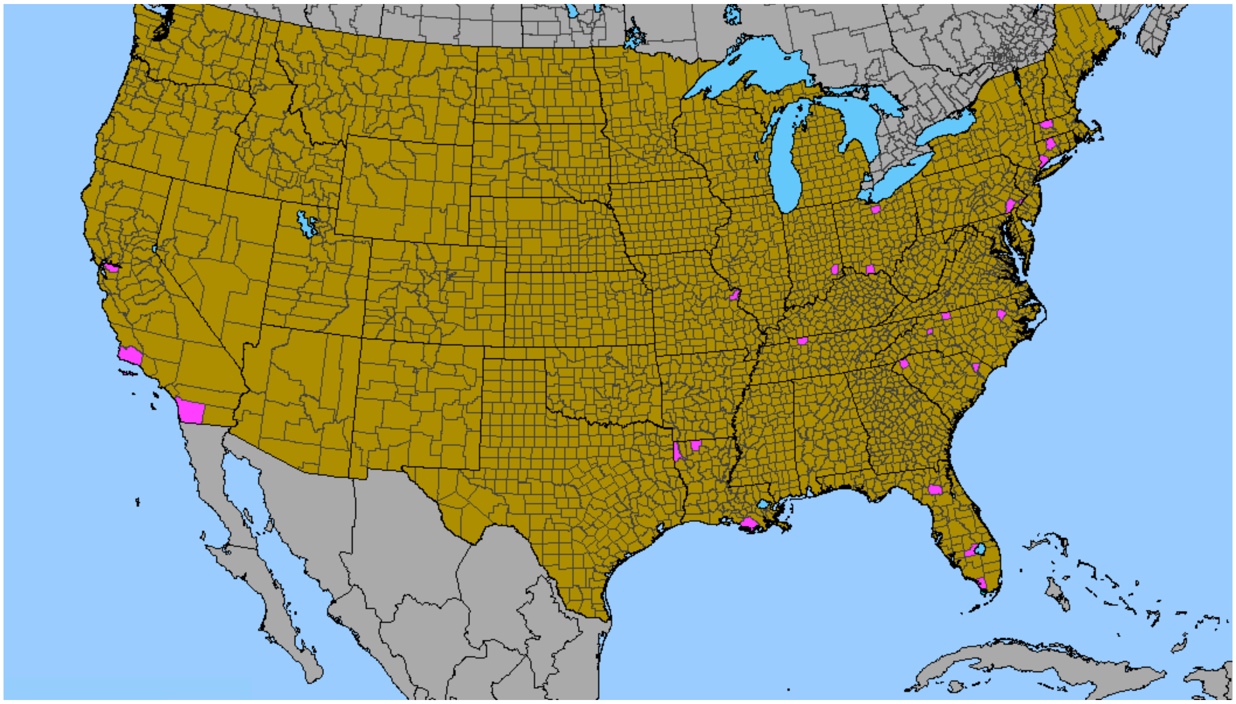

Figure S6. Map adapted from Kartesz (2015) showing the county distribution of *Nicotiana tabacum* (*NTA*). Magenta indicates that the species is present and non-native. Gray and brown represent no data and that the species is not present, respectively.

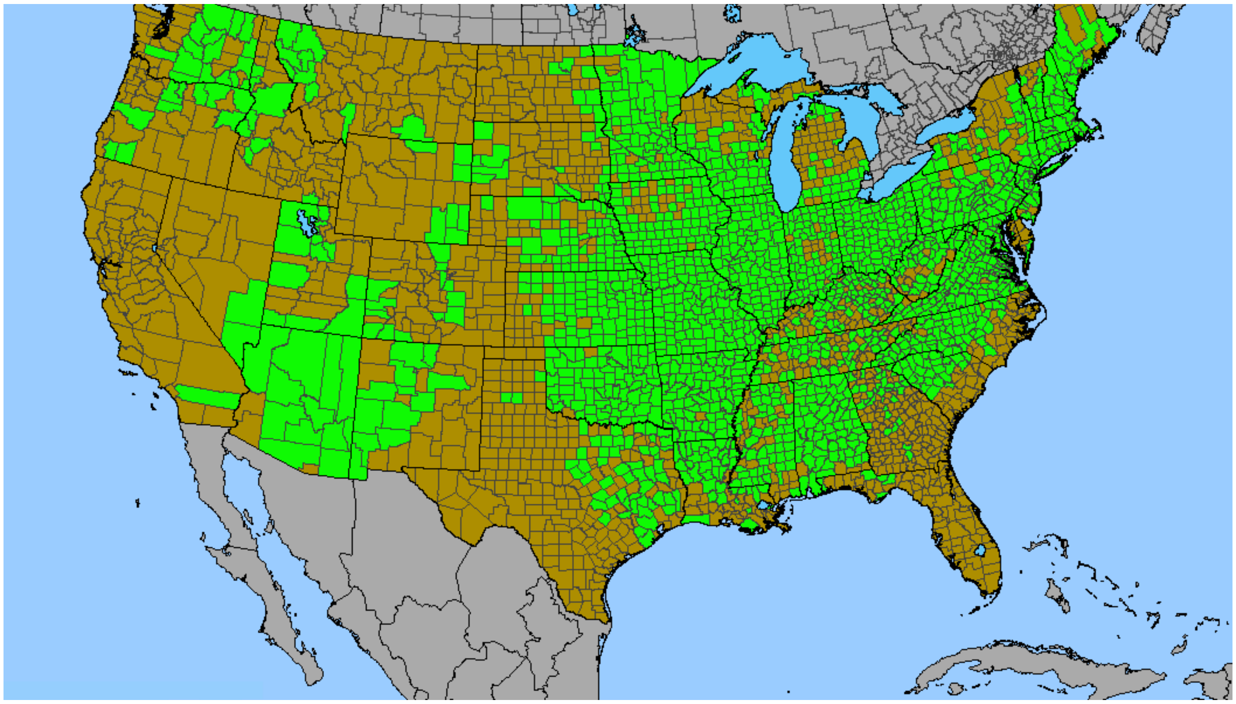

Figure S7. Map adapted from Kartesz (2015) showing the county distribution of *Rhus glabra* (*RGL*). Green indicates that the species is present and native. Gray and brown represent no data and that the species is not present, respectively.

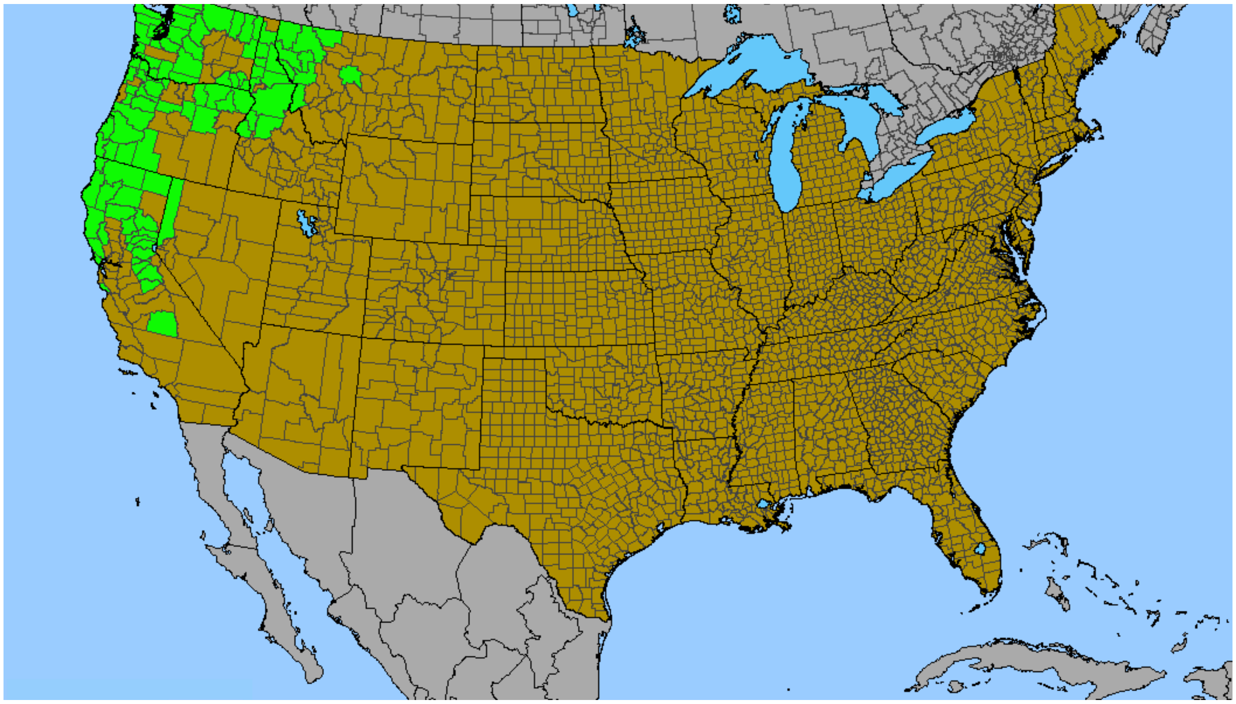

Figure S8. Map adapted from Kartesz (2015) showing the county distribution of *Taxus brevifolia* (*TBR*). Green indicates that the species is present and native. Gray and brown represent no data and that the species is not present, respectively.

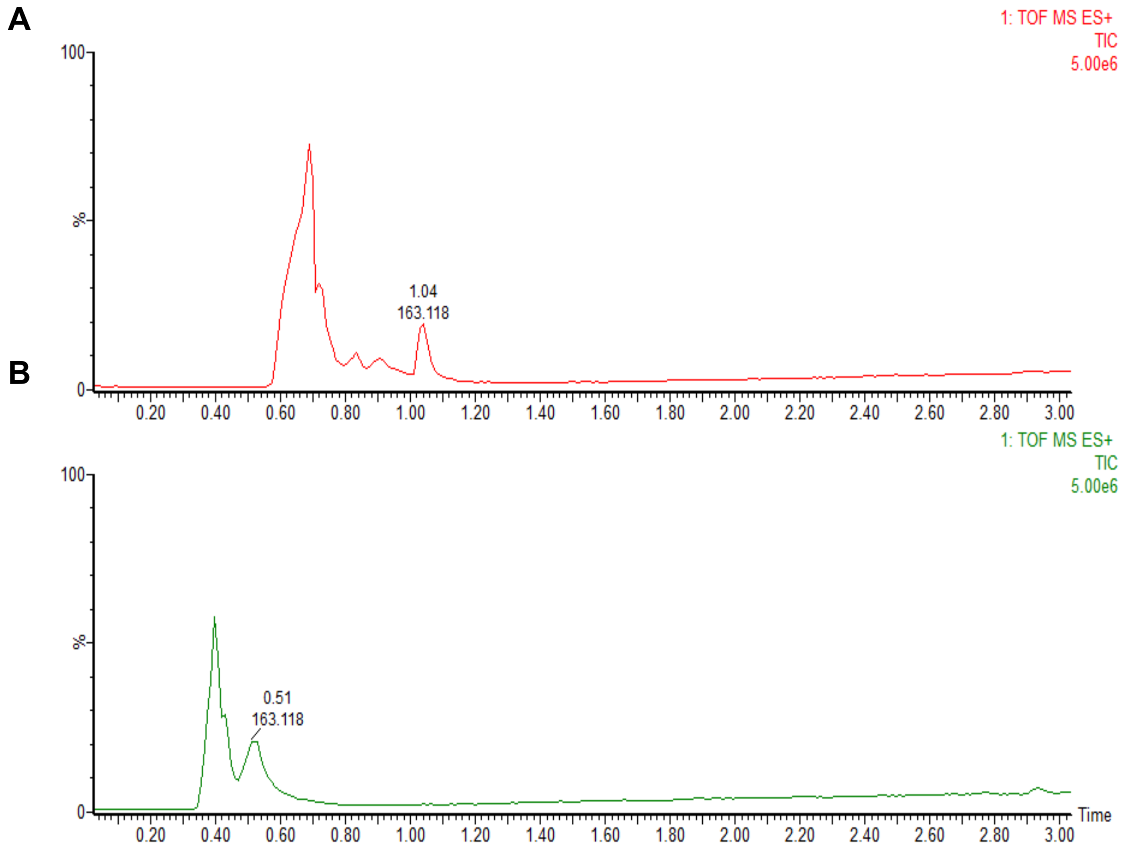

Figure S9. LC-MS total ion chromatogram (TIC) showing the retention of nicotine (163.118 *m/z*) on T3 **(A)** and C18 **(B)** columns. Nicotine had been detected in an experimental pipe smoked with *Nicotiana rustica* (*NRU*).

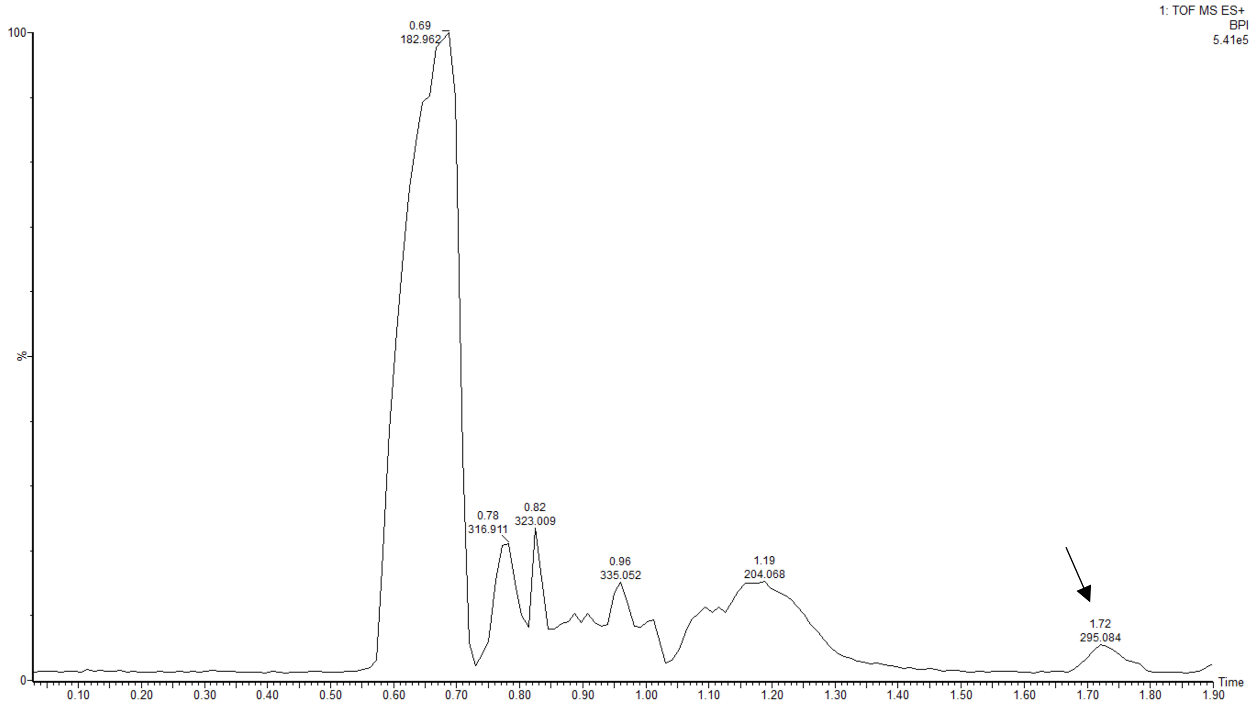

Figure S10. LC-MS base peak intensity (BPI) chromatogram of the *Arctostaphylos uva-ursi* (*AUV*) 2% aqueous tartaric acid (TA) quality control (QC) standard. The black arrow indicates where arbutin (295.084 *m/z*) was detected.

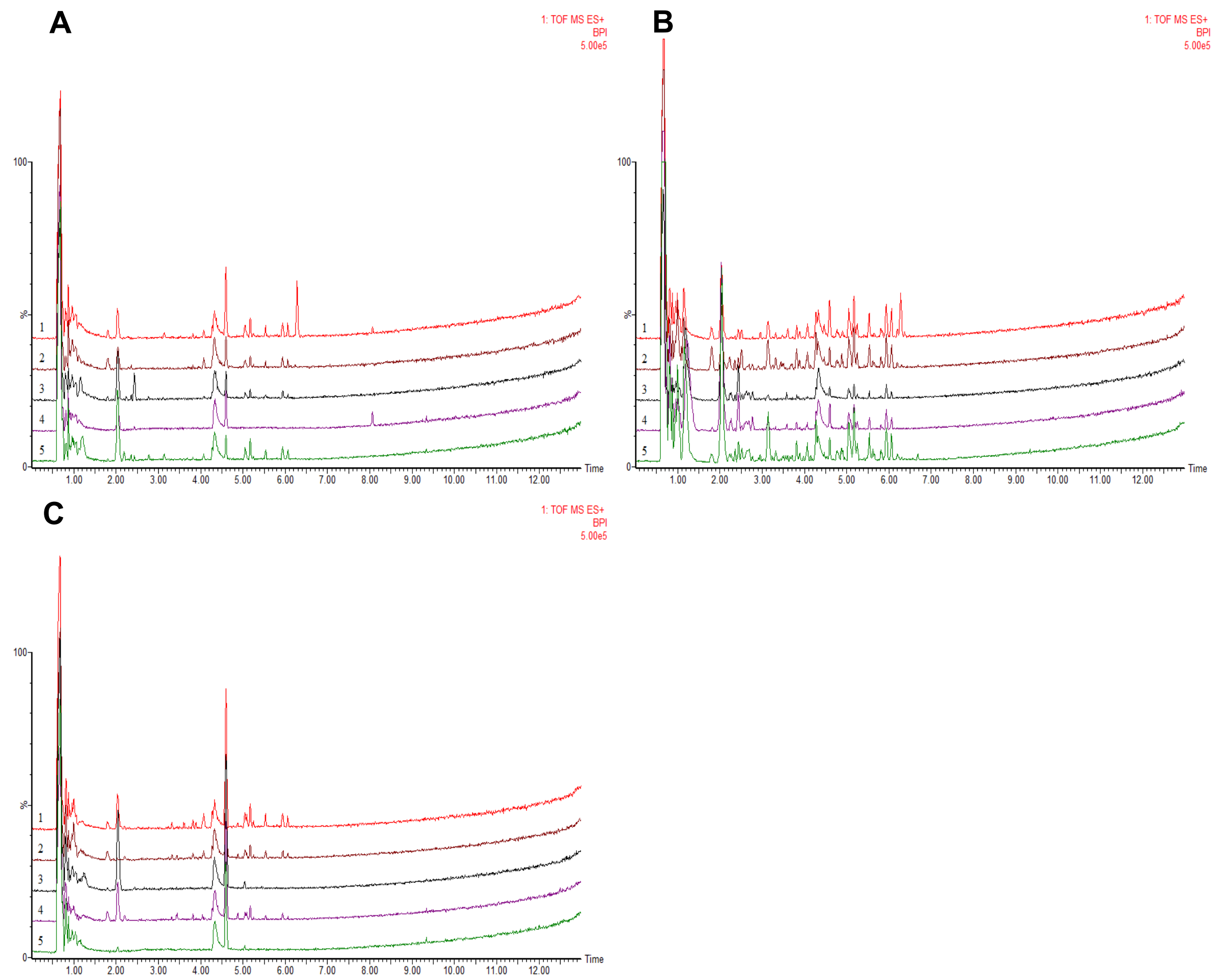

Figure S11. Each LC-MS base peak intensity (BPI) chromatogram shows five replicates (overlaid) of experimental pipes smoked with *Arctostaphylos uva-ursi* (*AUV*). The tip **(A)**, stem **(B)**, and bowl **(C)** sections were extracted with 2% aqueous tartaric acid (TA).

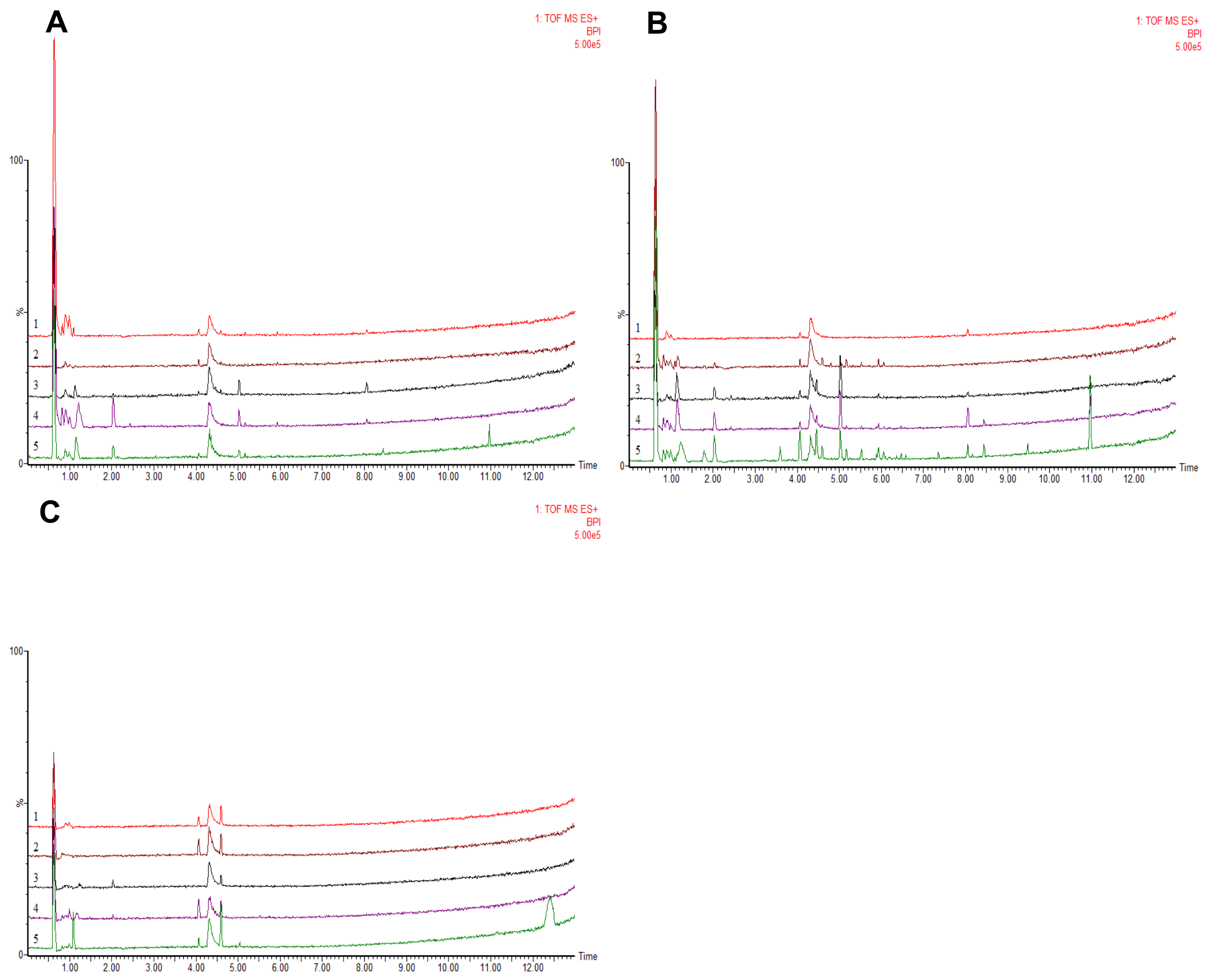

Figure S12. Each LC-MS base peak intensity (BPI) chromatogram shows five replicates (overlaid) of experimental pipes smoked with *Arctostaphylos uva-ursi* (*AUV*). The tip **(A)**, stem **(B)**, and bowl **(C)** sections were extracted with acetonitrile:2-propanol:water [3:2:2] (APW).

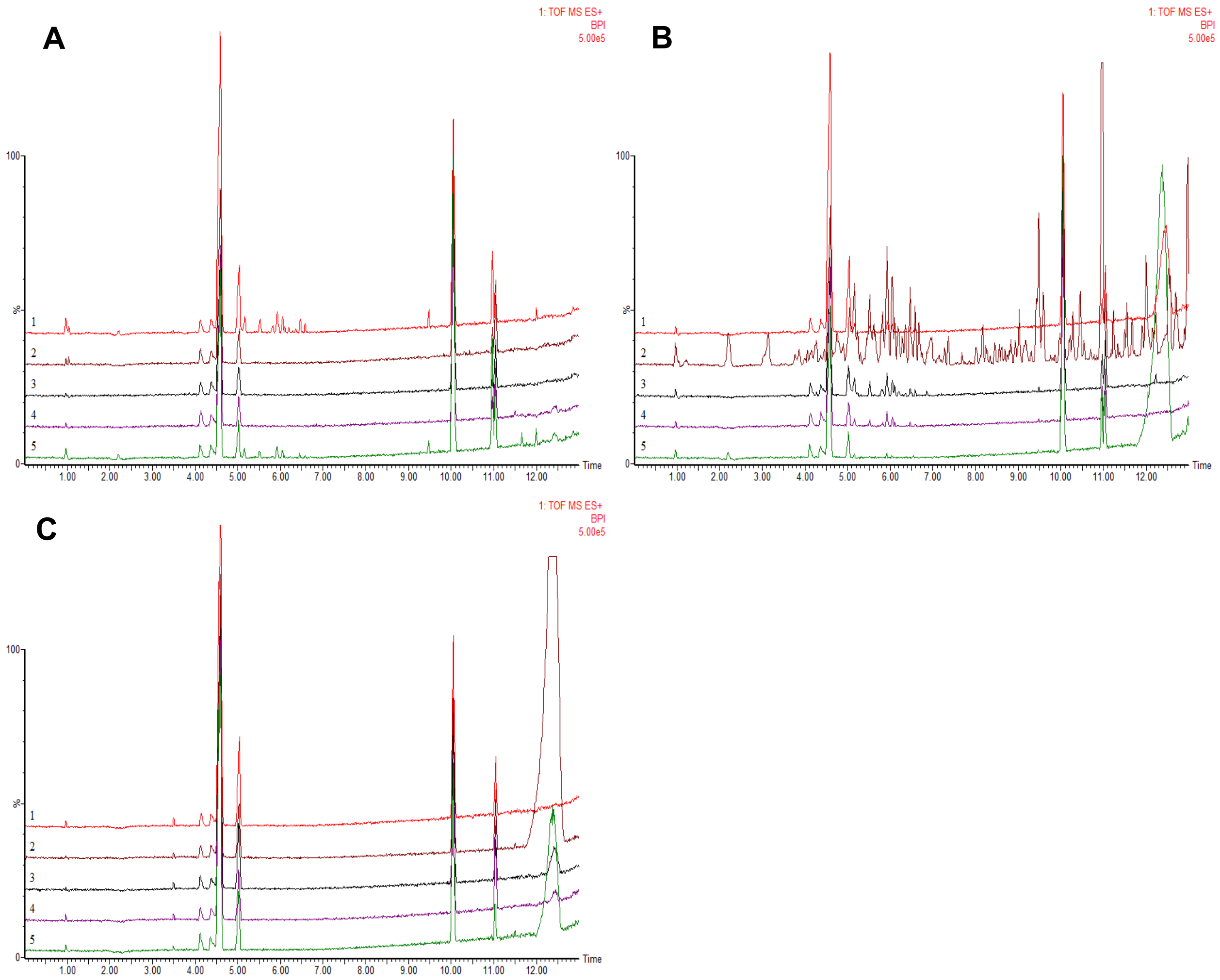

Figure S13. Each LC-MS base peak intensity (BPI) chromatogram shows five replicates (overlaid) of experimental pipes smoked with *Arctostaphylos uva-ursi* (*AUV*). The tip **(A)**, stem **(B)**, and bowl **(C)** sections were extracted with methyl *tert*-butyl ether (MTBE).

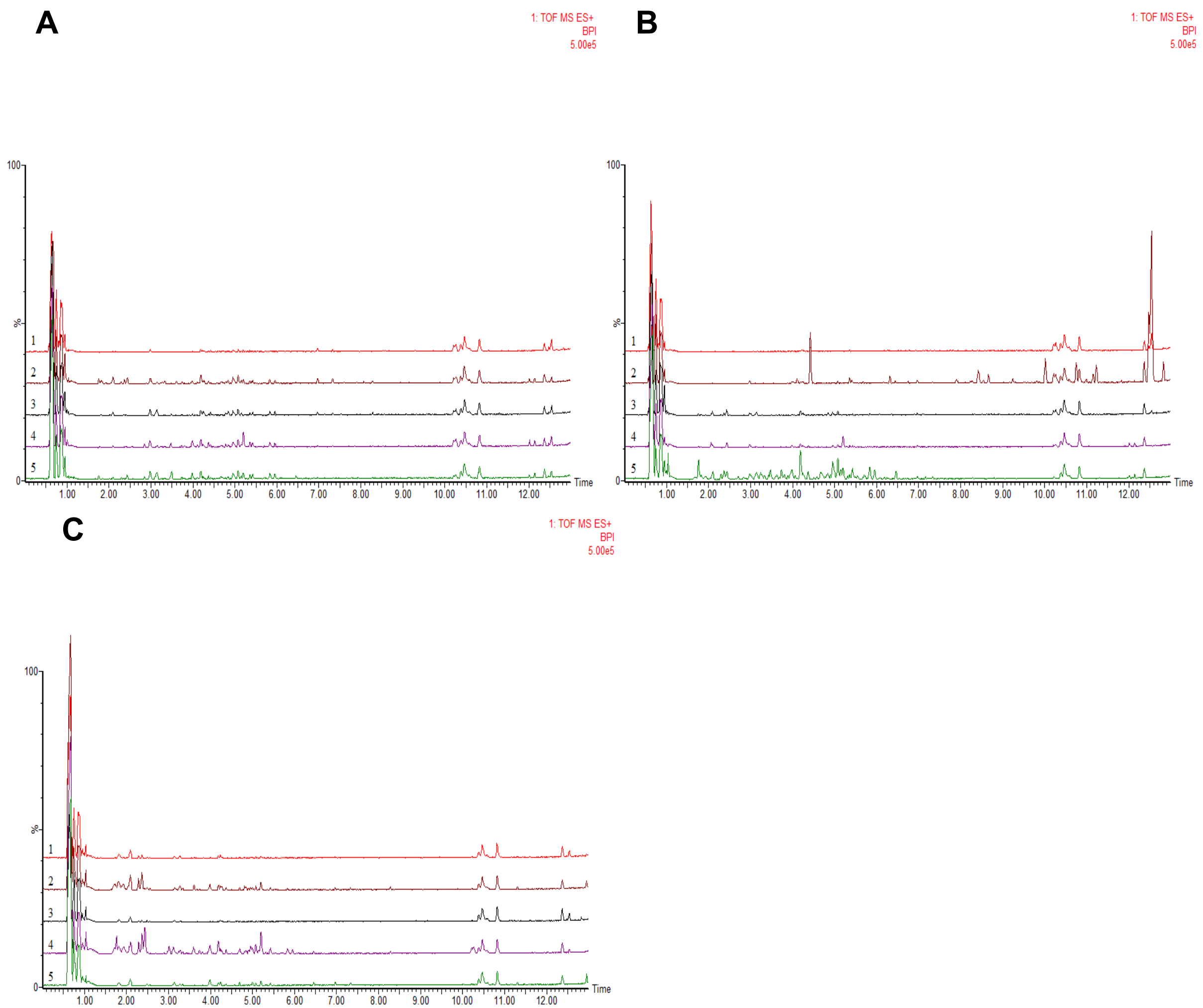

Figure S14. Each LC-MS base peak intensity (BPI) chromatogram shows five replicates (overlaid) of experimental pipes smoked with *Cornus sericea* (*CSE*). The tip **(A)**, stem **(B)**, and bowl **(C)** sections were extracted with 2% aqueous tartaric acid (TA).

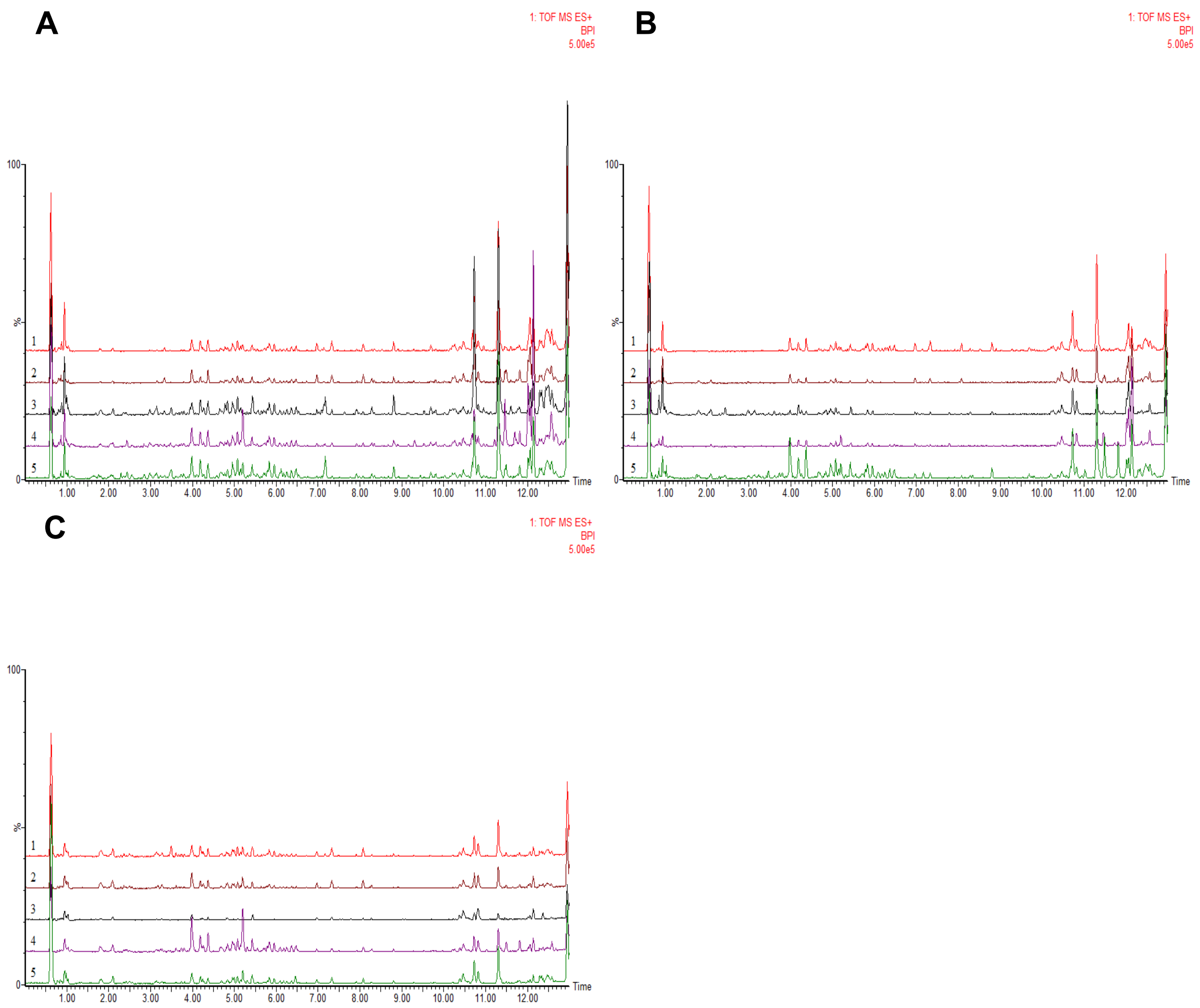

Figure S15. Each LC-MS base peak intensity (BPI) chromatogram shows five replicates (overlaid) of experimental pipes smoked with *Cornus sericea* (*CSE*). The tip **(A)**, stem **(B)**, and bowl **(C)** sections were extracted with acetonitrile:2-propanol:water [3:2:2] (APW).

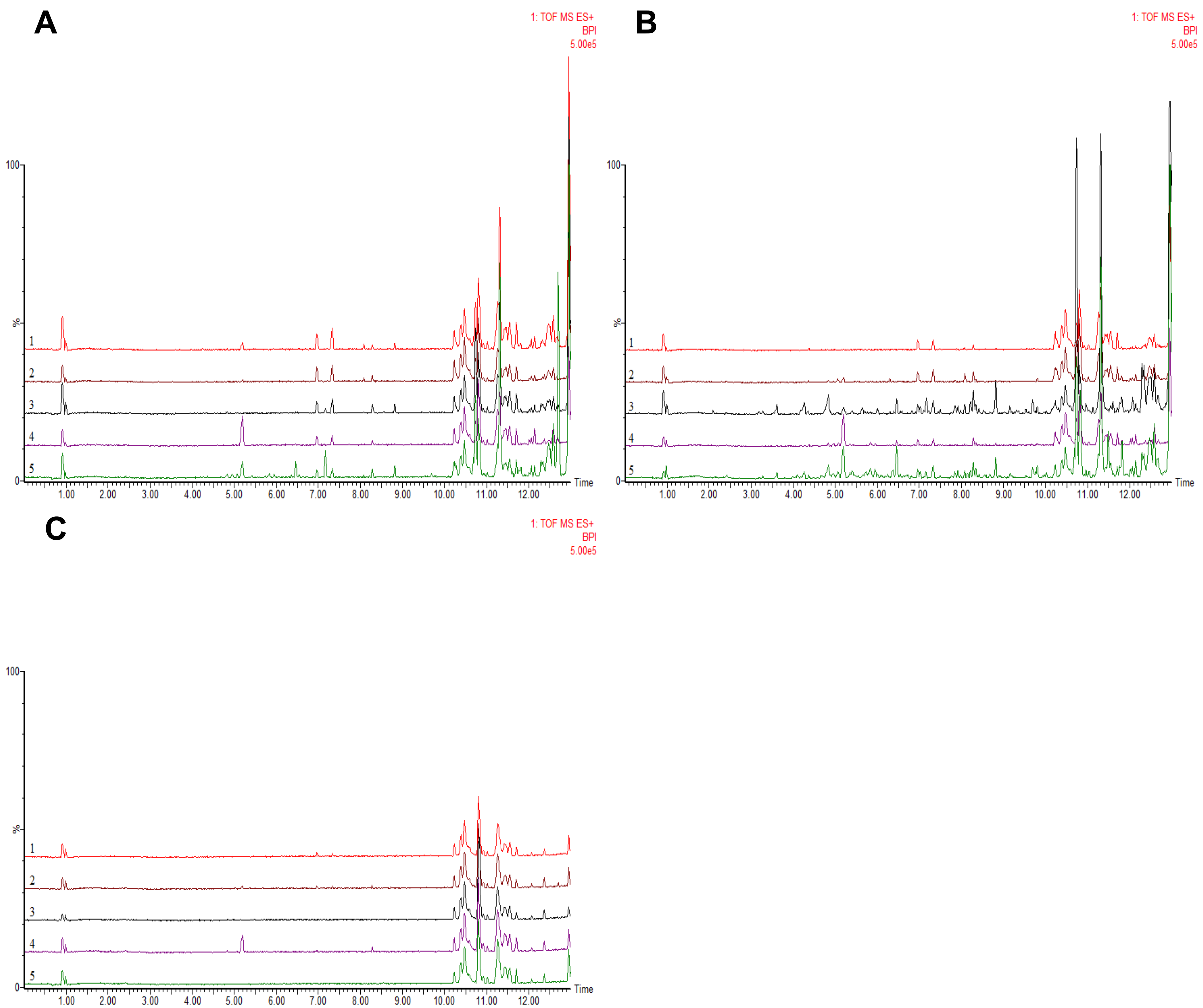

Figure S16. Each LC-MS base peak intensity (BPI) chromatogram shows five replicates (overlaid) of experimental pipes smoked with *Cornus sericea* (*CSE*). The tip **(A)**, stem **(B)**, and bowl **(C)** sections were extracted with methyl *tert*-butyl ether (MTBE).

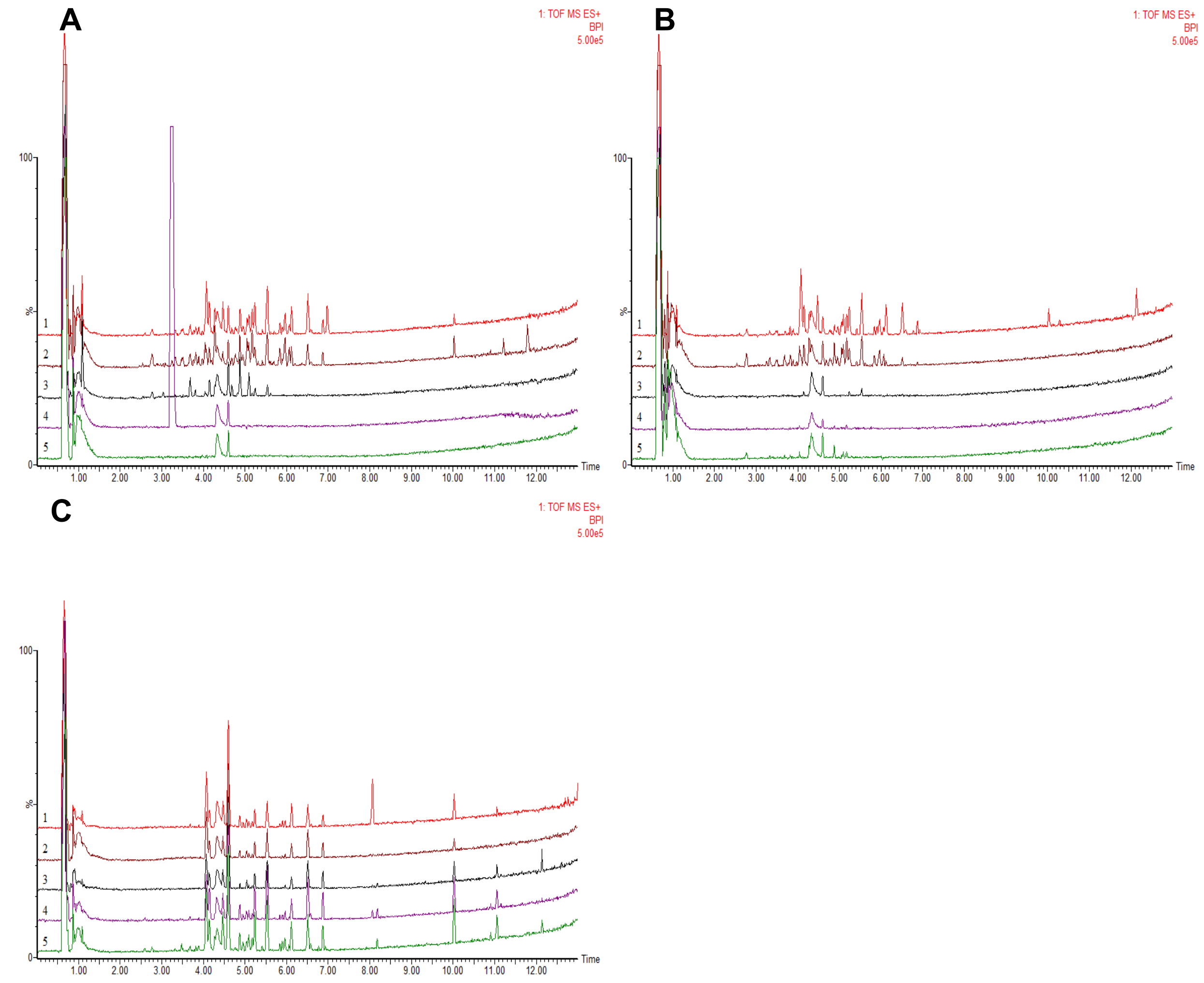

Figure S17. Each LC-MS base peak intensity (BPI) chromatogram shows five replicates (overlaid) of experimental pipes smoked with *Nicotiana attenuata* (*NAT*). The tip **(A)**, stem **(B)**, and bowl **(C)** sections were extracted with 2% aqueous tartaric acid (TA).

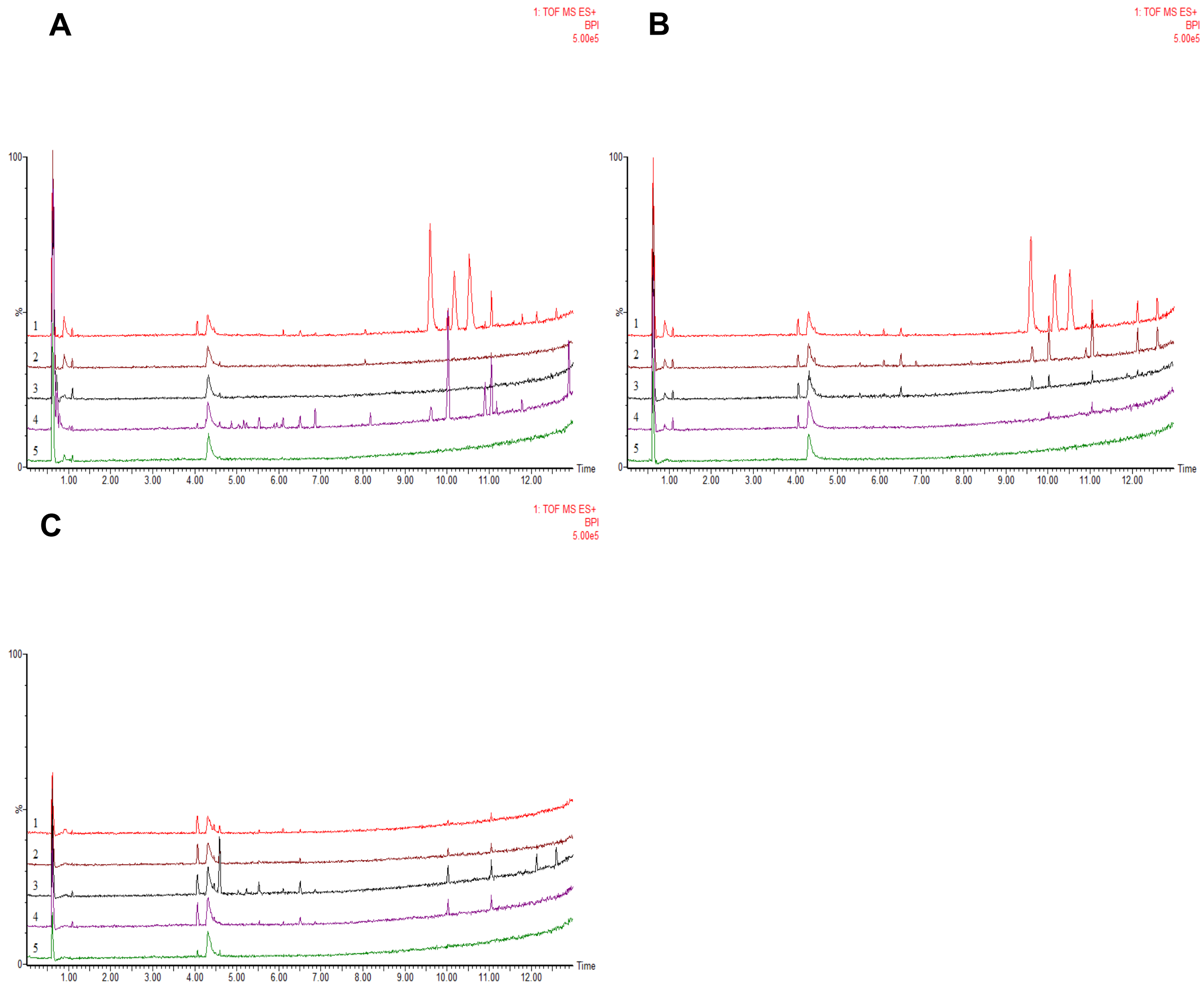

Figure S18. Each LC-MS base peak intensity (BPI) chromatogram shows five replicates (overlaid) of experimental pipes smoked with *Nicotiana attenuata* (*NAT*). The tip **(A)**, stem **(B)**, and bowl **(C)** sections were extracted with acetonitrile:2-propanol:water [3:2:2] (APW).

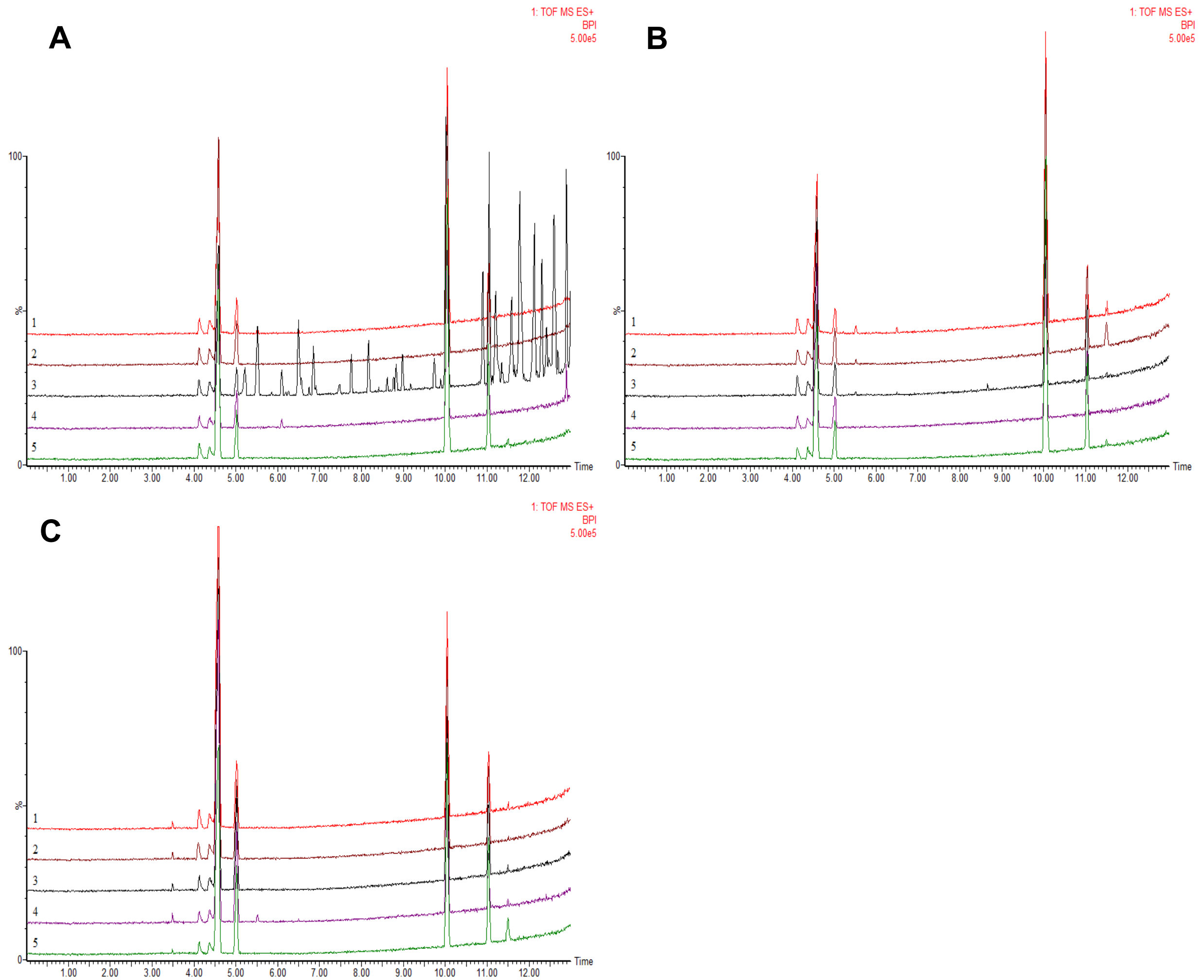

Figure S19. Each LC-MS base peak intensity (BPI) chromatogram shows five replicates (overlaid) of experimental pipes smoked with *Nicotiana attenuata* (*NAT*). The tip **(A)**, stem **(B)**, and bowl **(C)** sections were extracted with methyl *tert*-butyl ether (MTBE).

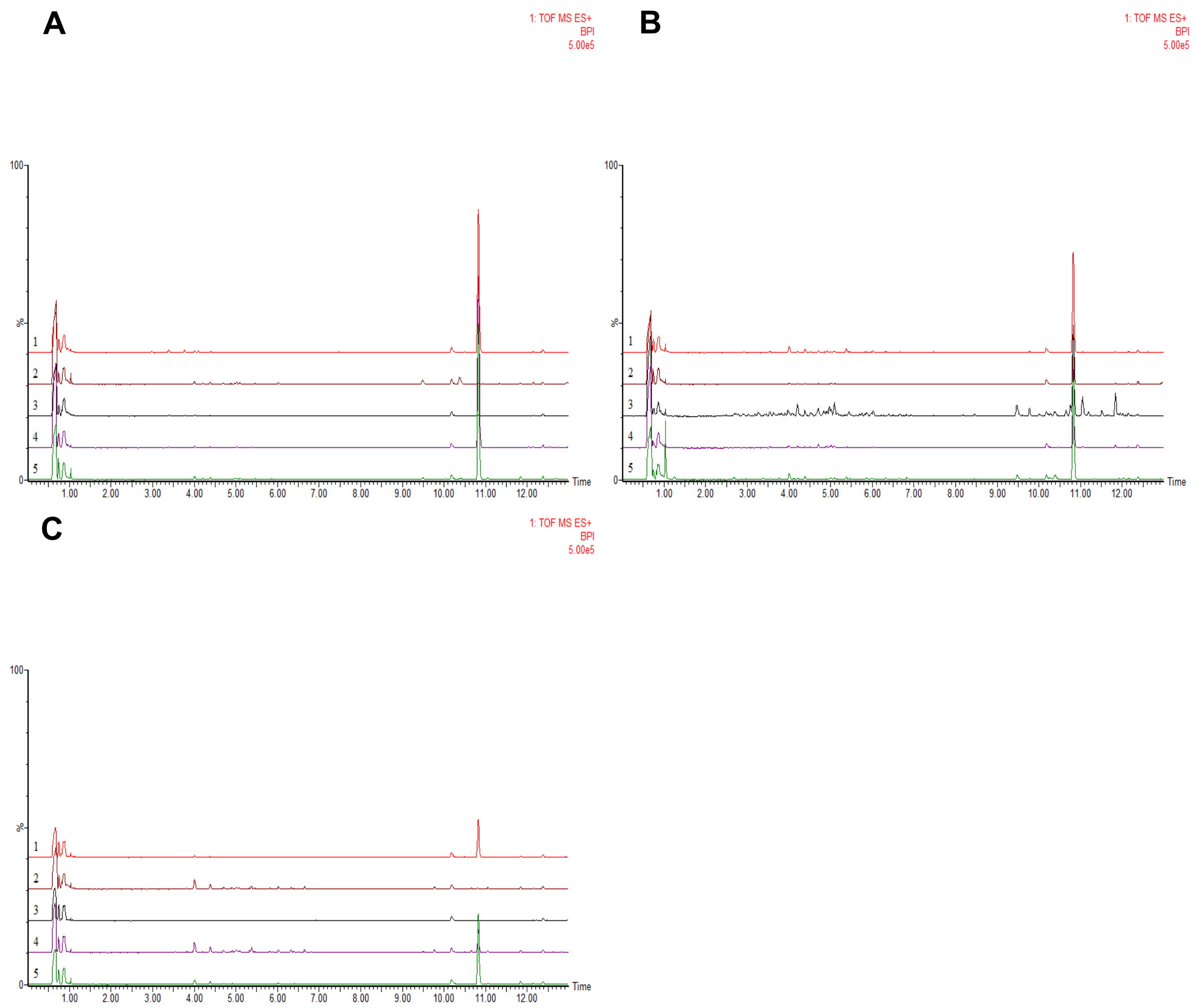

Figure S20. Each LC-MS base peak intensity (BPI) chromatogram shows five replicates (overlaid) of experimental pipes smoked with *Nicotiana quadrivalvis* (*NQU*). The tip **(A)**, stem **(B)**, and bowl **(C)** sections were extracted with 2% aqueous tartaric acid (TA).

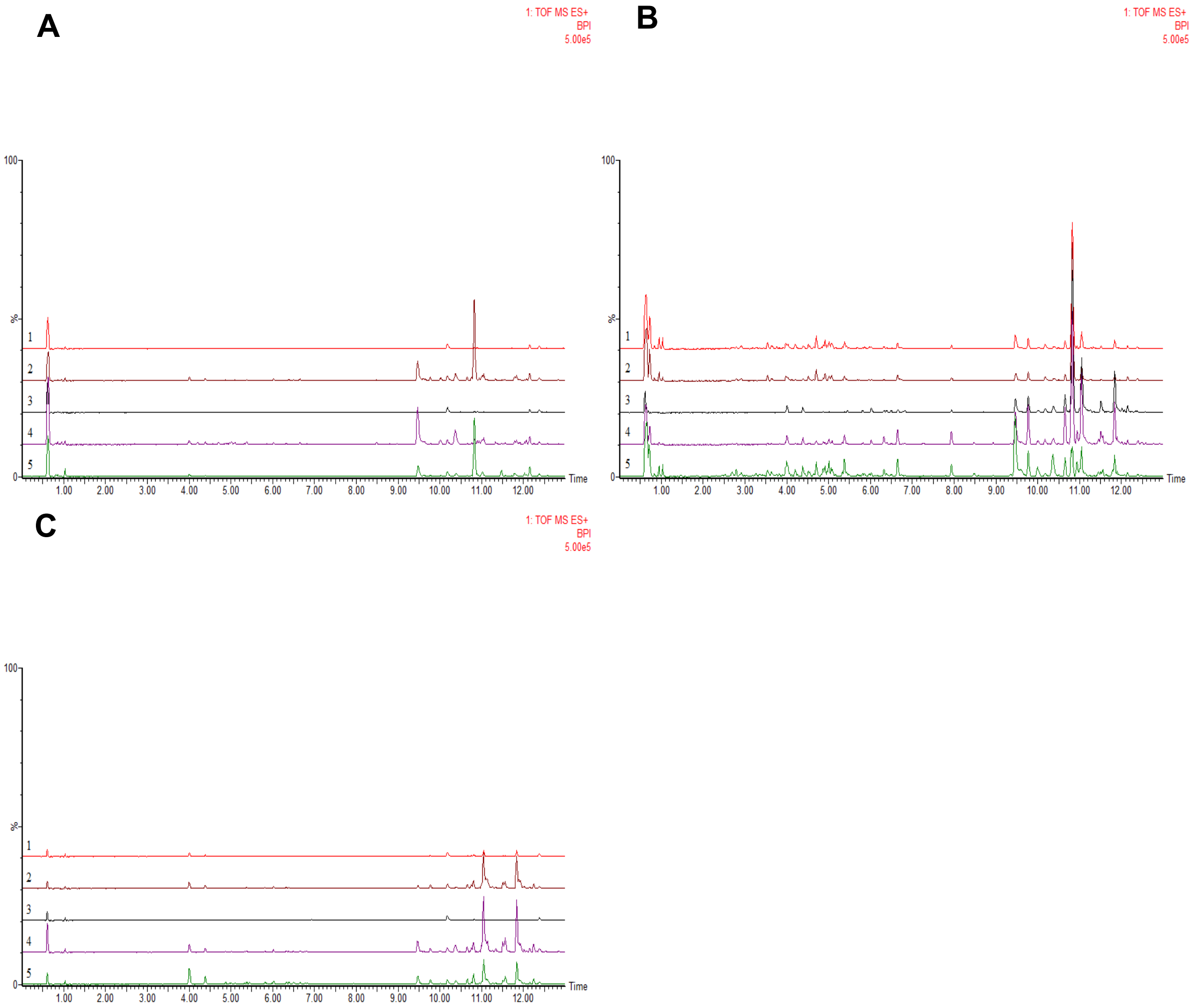

Figure S21. Each LC-MS base peak intensity (BPI) chromatogram shows five replicates (overlaid) of experimental pipes smoked with *Nicotiana quadrivalvis* (*NQU*). The tip **(A)**, stem **(B)**, and bowl **(C)** sections were extracted with acetonitrile:2-propanol:water [3:2:2] (APW).

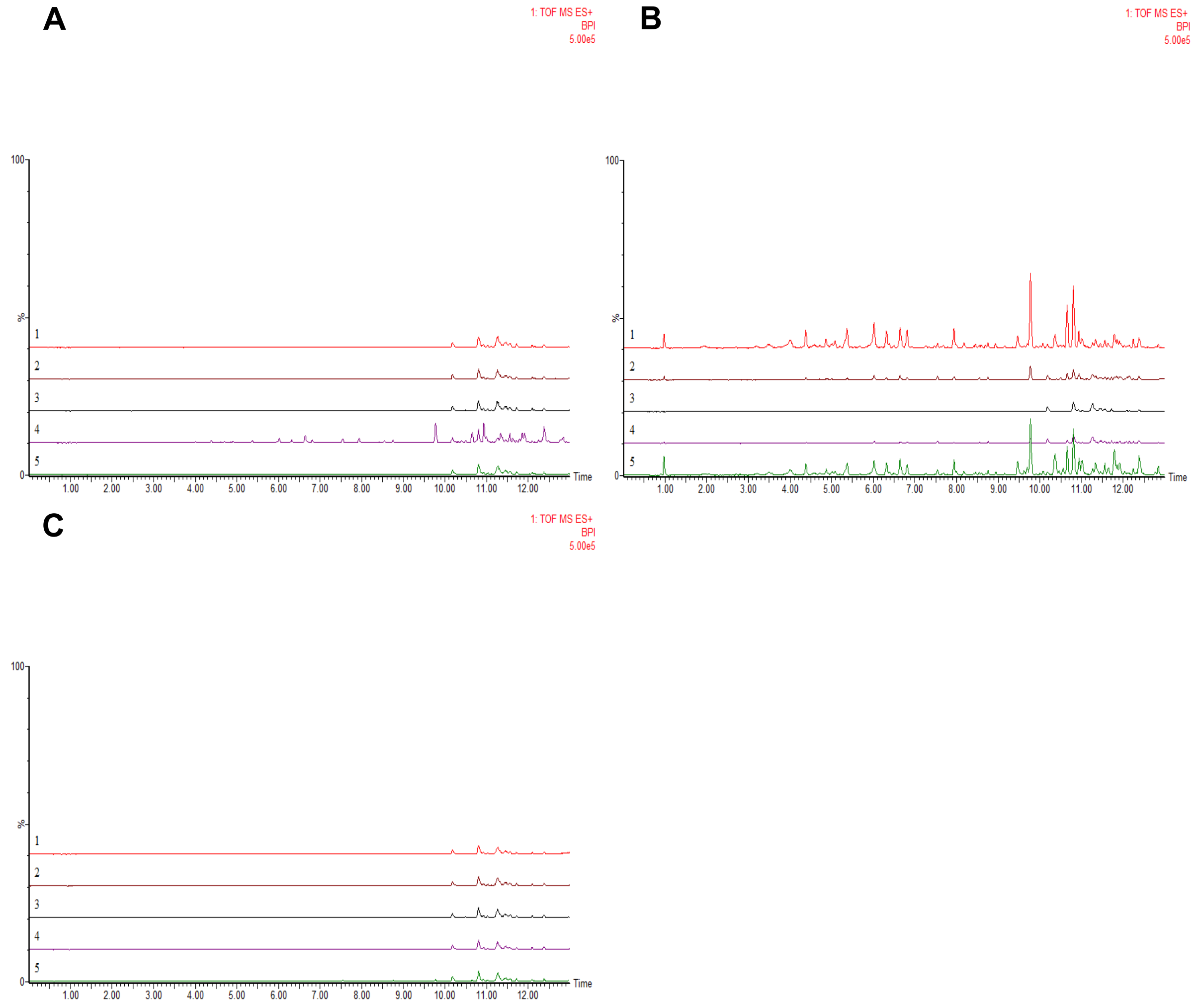

Figure S22. Each LC-MS base peak intensity (BPI) chromatogram shows five replicates (overlaid) of experimental pipes smoked with *Nicotiana quadrivalvis* (*NQU*). The tip **(A)**, stem **(B)**, and bowl **(C)** sections were extracted with methyl *tert*-butyl ether (MTBE).

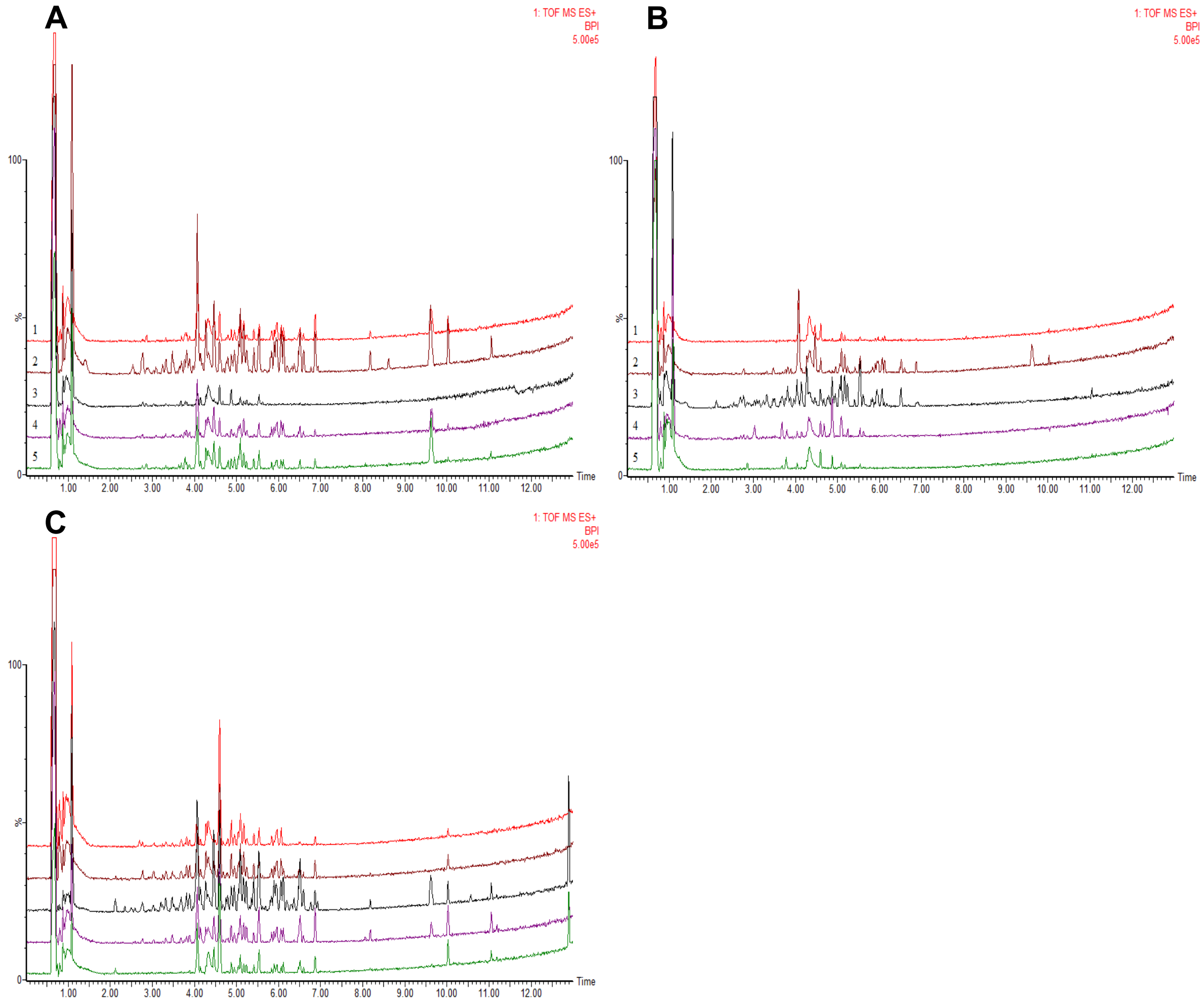

Figure S23. Each LC-MS base peak intensity (BPI) chromatogram shows five replicates (overlaid) of experimental pipes smoked with *Nicotiana rustica* (*NRU*). The tip **(A)**, stem **(B)**, and bowl **(C)** sections were extracted with 2% aqueous tartaric acid (TA).

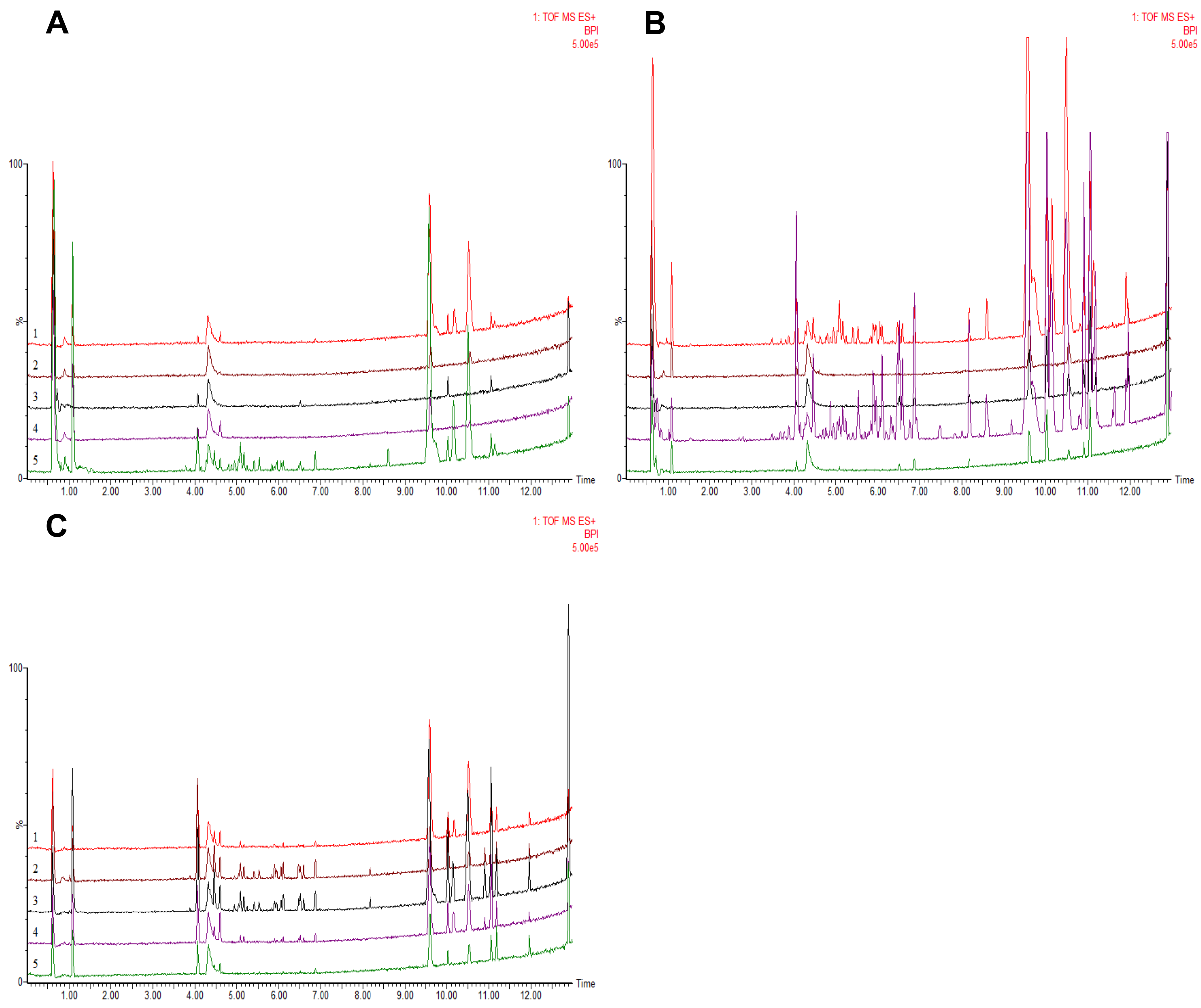

Figure S24. Each LC-MS base peak intensity (BPI) chromatogram shows five replicates (overlaid) of experimental pipes smoked with *Nicotiana rustica* (*NRU*). The tip **(A)**, stem **(B)**, and bowl **(C)** sections were extracted with acetonitrile:2-propanol:water [3:2:2] (APW).

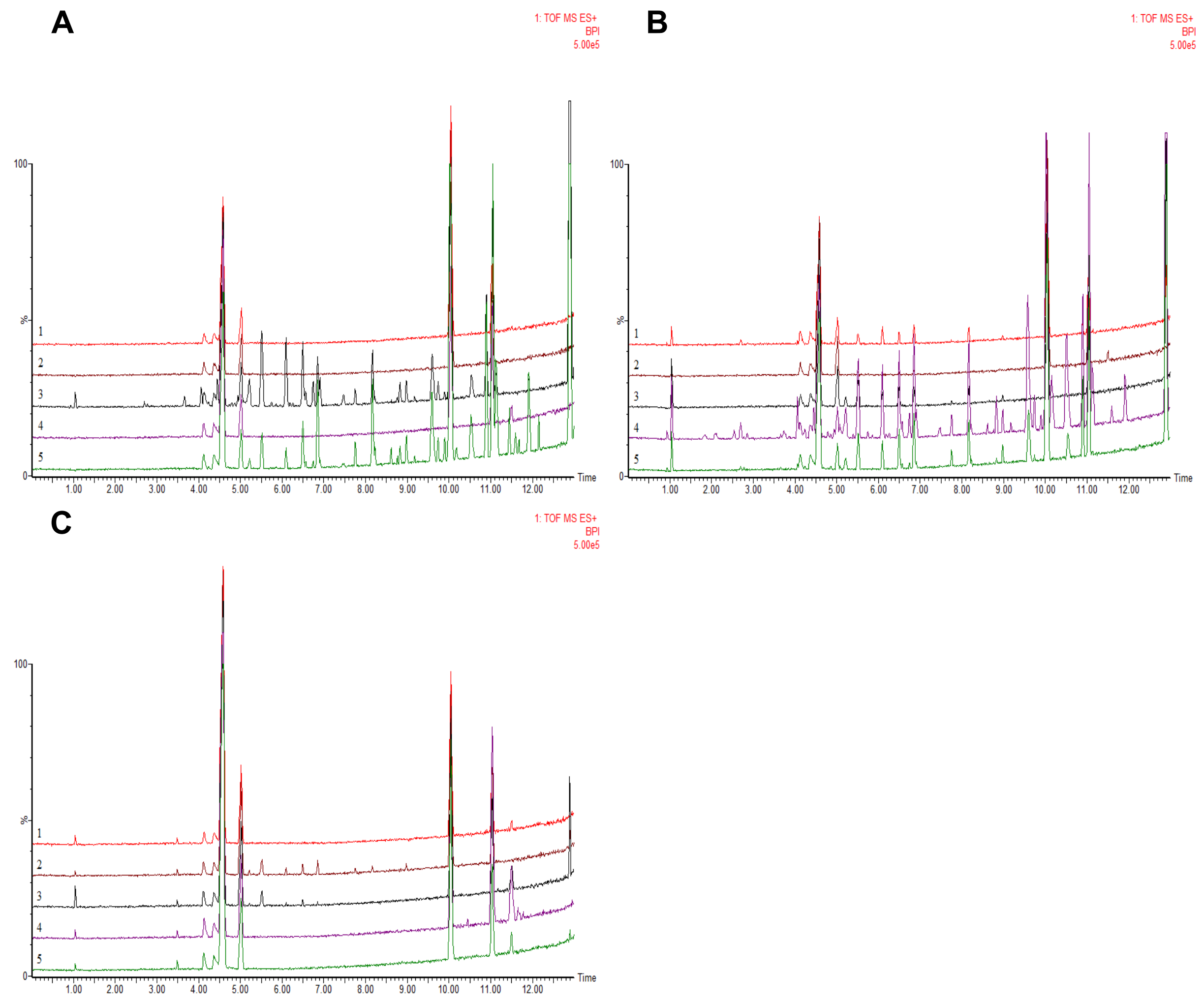

Figure S25. Each LC-MS base peak intensity (BPI) chromatogram shows five replicates (overlaid) of experimental pipes smoked with *Nicotiana rustica* (*NRU*). The tip **(A)**, stem **(B)**, and bowl **(C)** sections were extracted with methyl *tert*-butyl ether (MTBE).

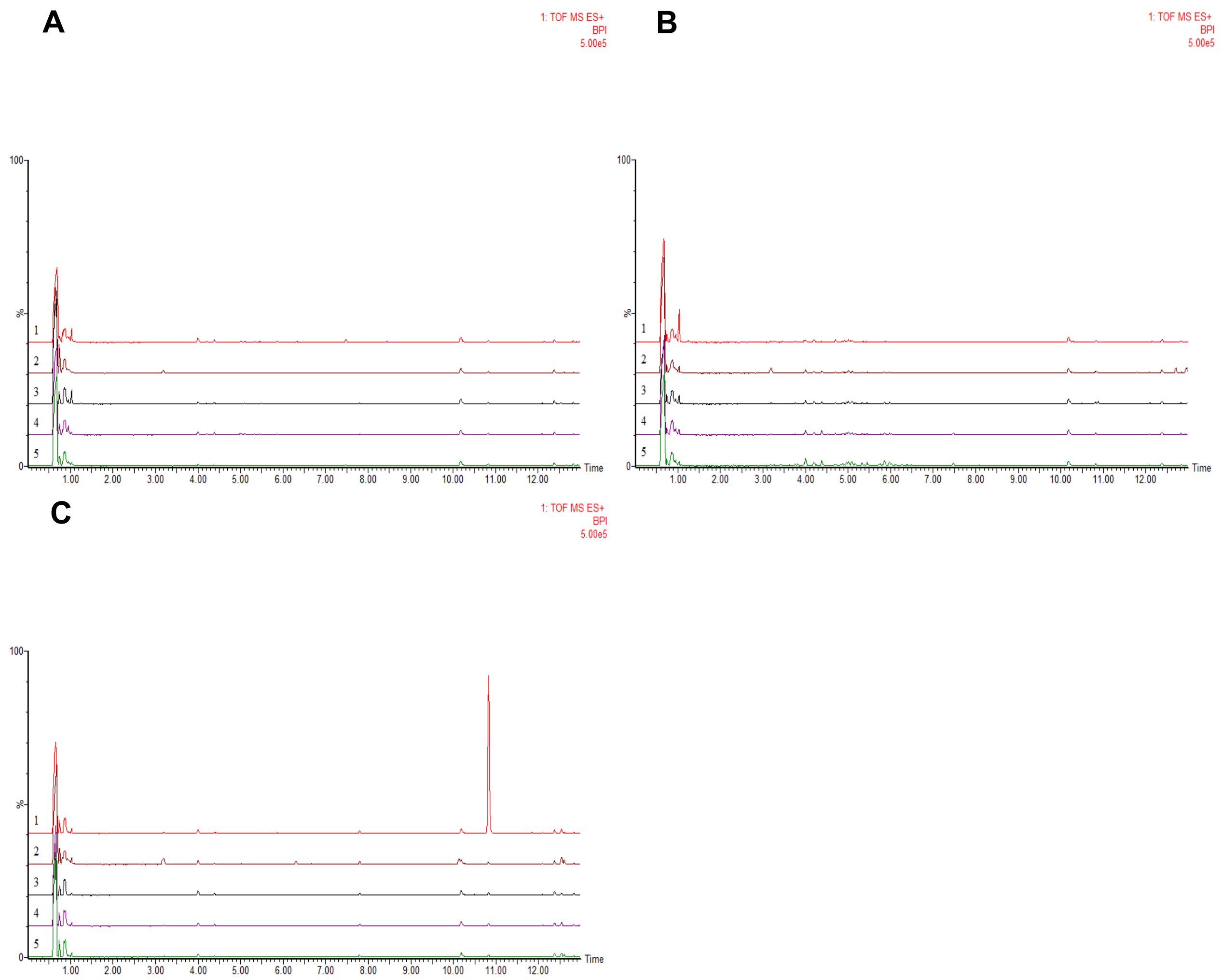

Figure S26. Each LC-MS base peak intensity (BPI) chromatogram shows five replicates (overlaid) of experimental pipes smoked with *Nicotiana tabacum* (*NTA*). The tip **(A)**, stem **(B)**, and bowl **(C)** sections were extracted with 2% aqueous tartaric acid (TA).

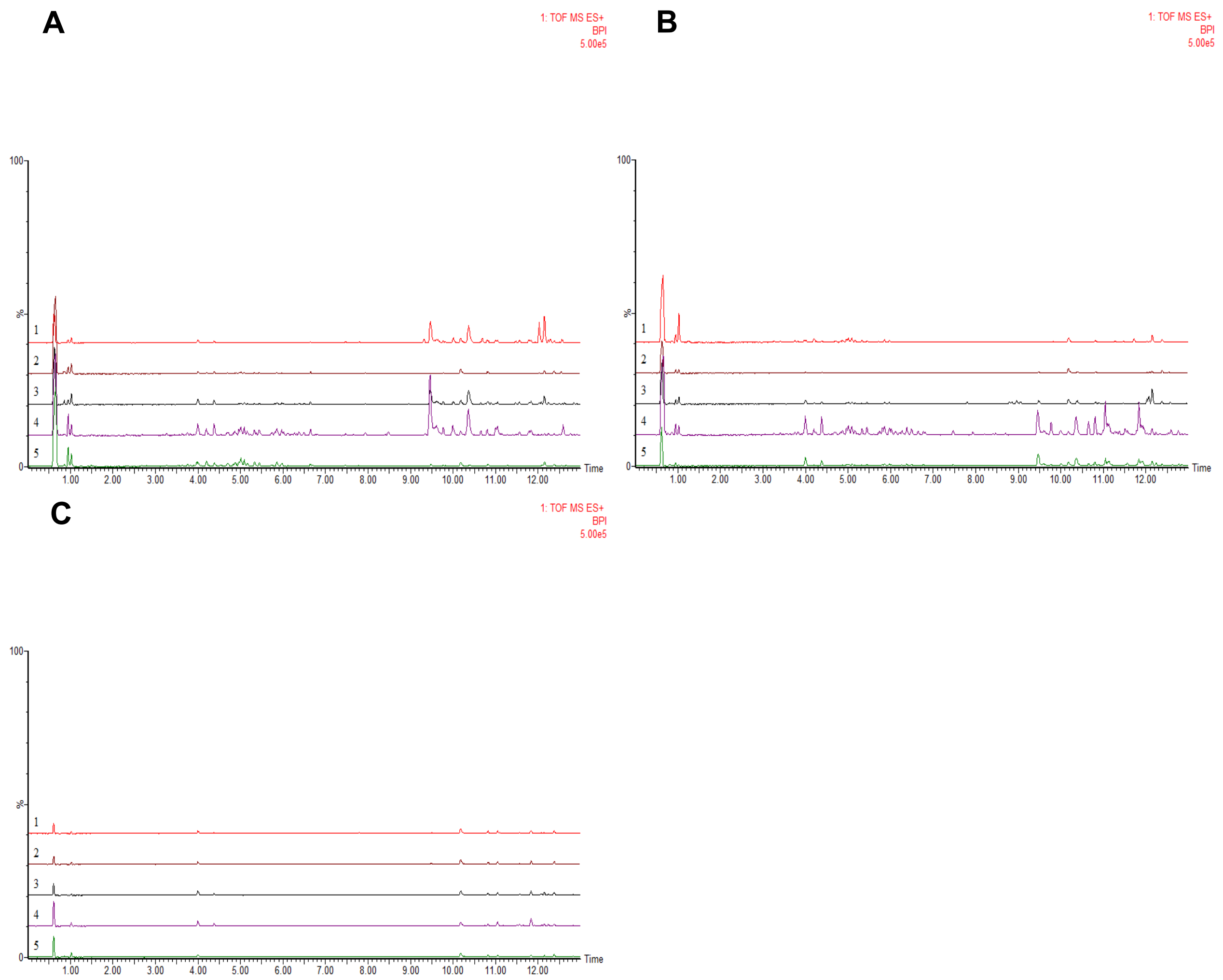

Figure S27. Each LC-MS base peak intensity (BPI) chromatogram shows five replicates (overlaid) of experimental pipes smoked with *Nicotiana tabacum* (*NTA*). The tip **(A)**, stem **(B)**, and bowl **(C)** sections were extracted with acetonitrile:2-propanol:water [3:2:2] (APW).

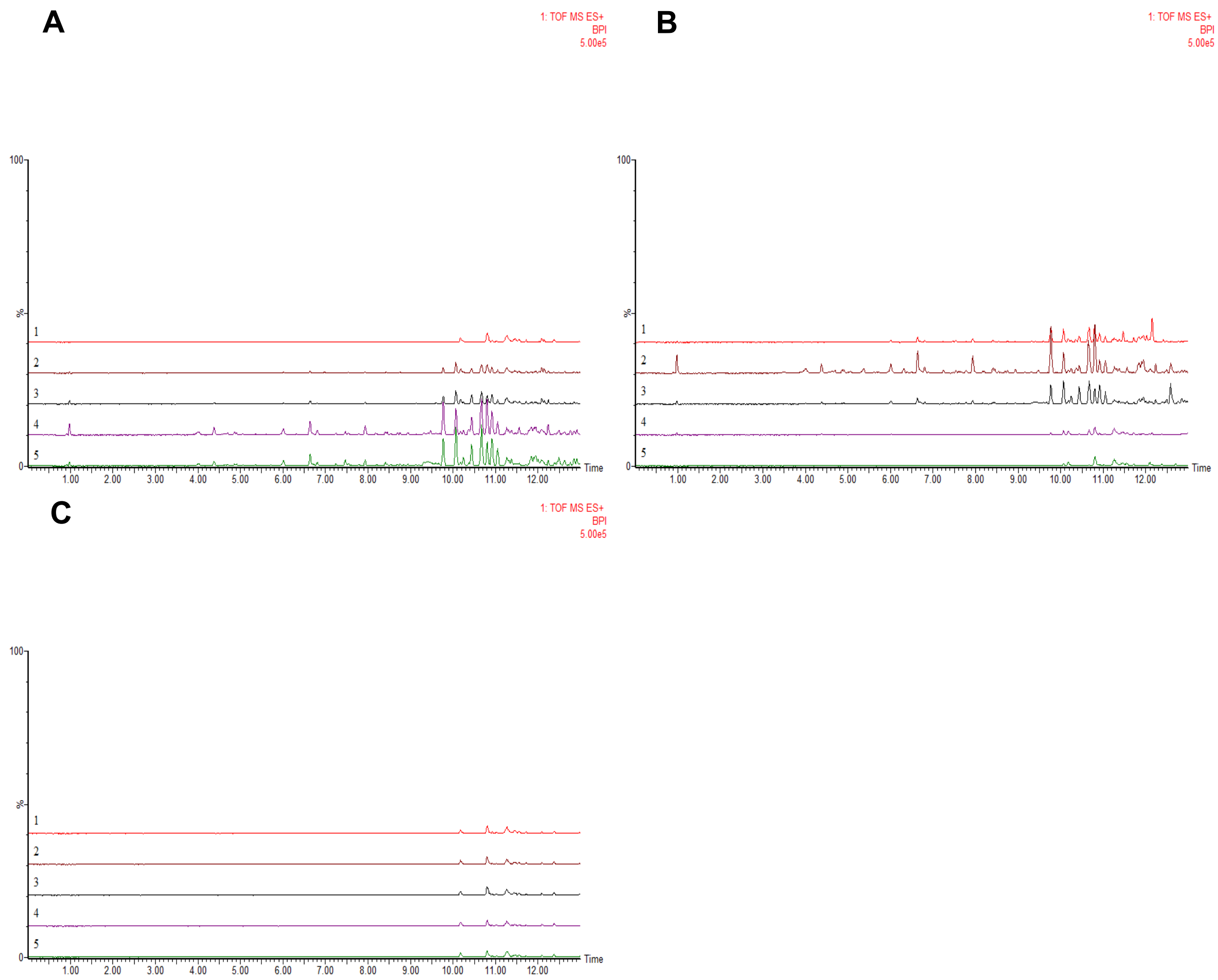

Figure S28. Each LC-MS base peak intensity (BPI) chromatogram shows five replicates (overlaid) of experimental pipes smoked with *Nicotiana tabacum* (*NTA*). The tip **(A)**, stem **(B)**, and bowl **(C)** sections were extracted with methyl *tert*-butyl ether (MTBE).

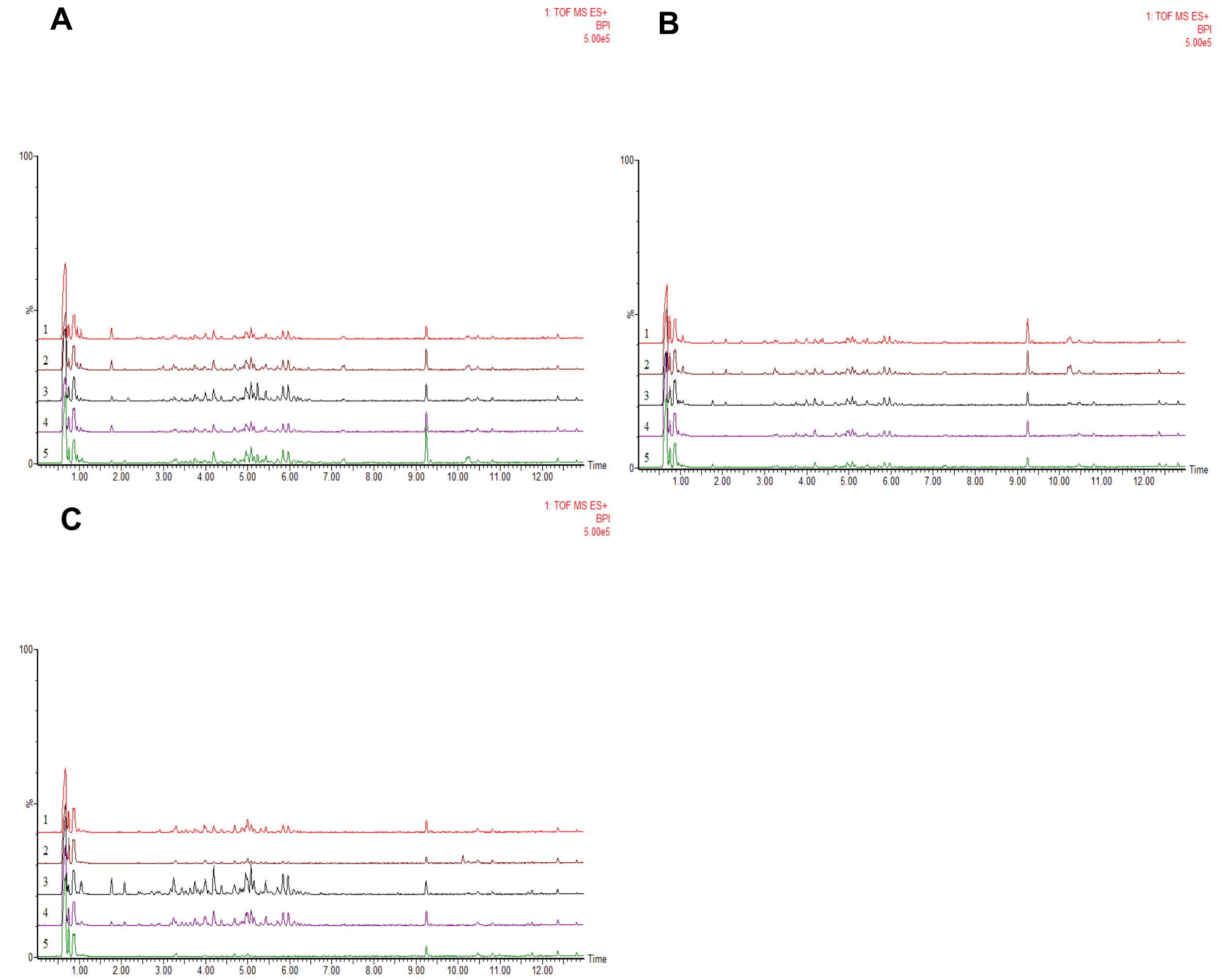

Figure S29. Each LC-MS base peak intensity (BPI) chromatogram shows five replicates (overlaid) of experimental pipes smoked with *Rhus glabra* (*RGL*). The tip **(A)**, stem **(B)**, and bowl **(C)** sections were extracted with 2% aqueous tartaric acid (TA).

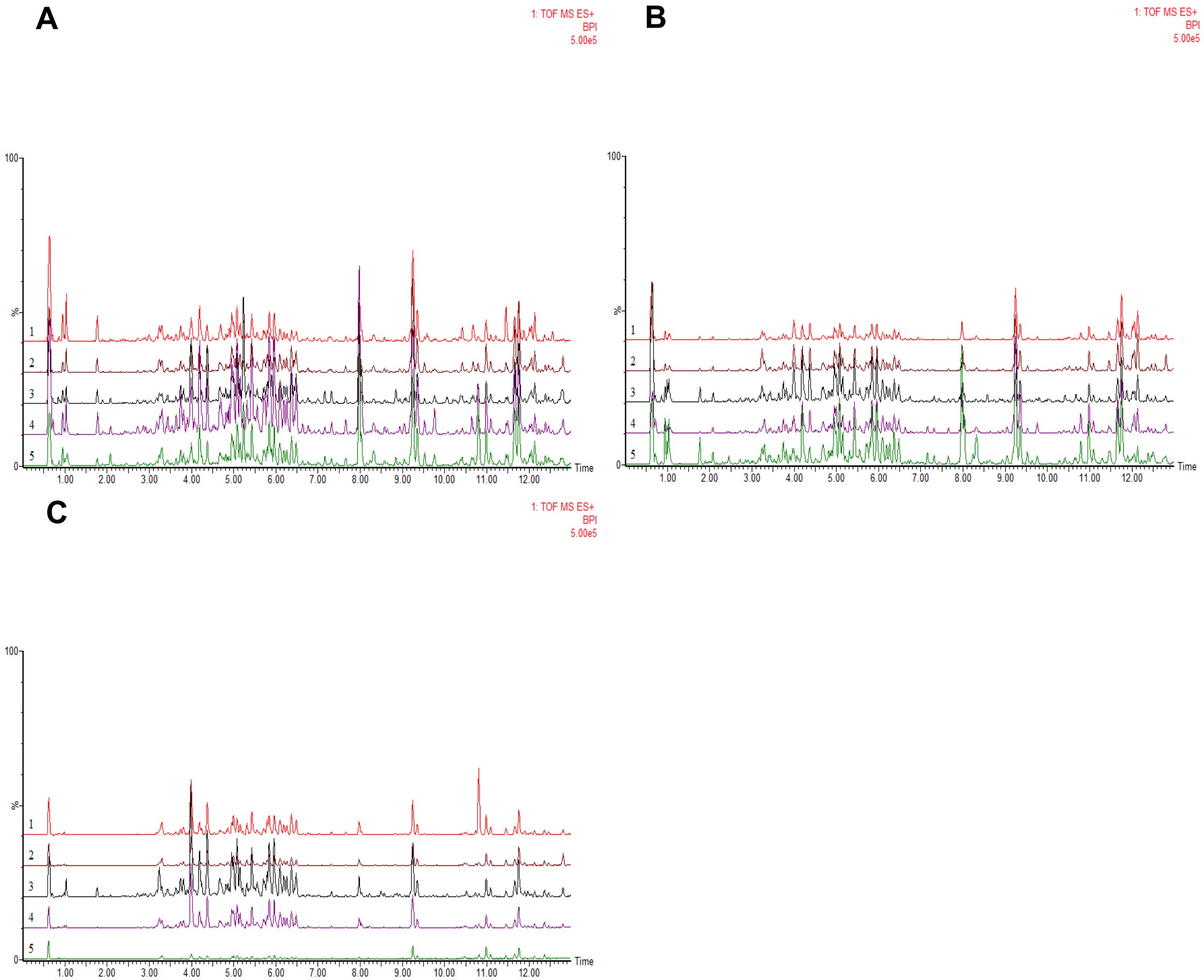

Figure S30. Each LC-MS base peak intensity (BPI) chromatogram shows five replicates (overlaid) of experimental pipes smoked with *Rhus glabra* (*RGL*). The tip **(A)**, stem **(B)**, and bowl **(C)** sections were extracted with acetonitrile:2-propanol:water [3:2:2] (APW).

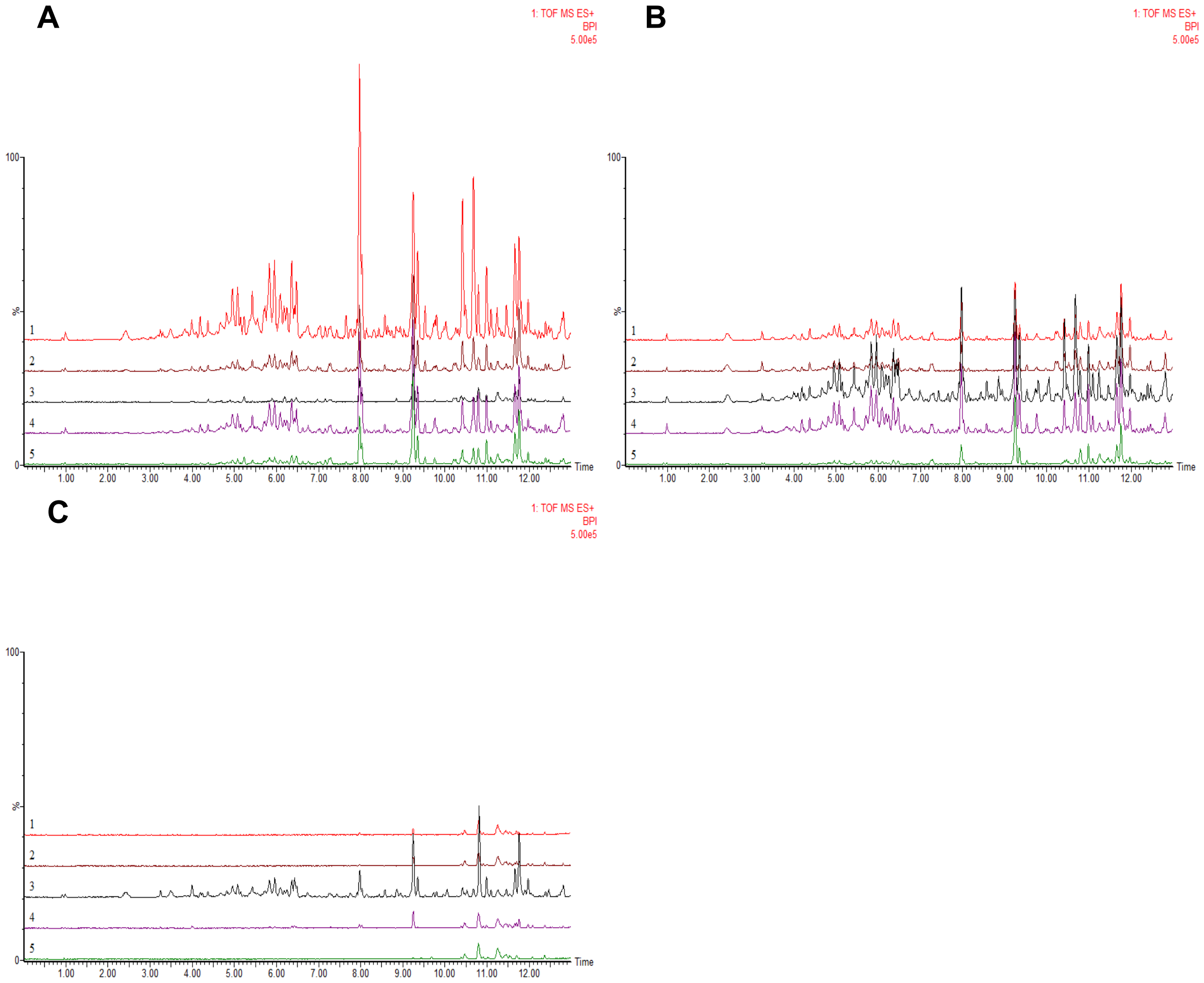

Figure S31. Each LC-MS base peak intensity (BPI) chromatogram shows five replicates (overlaid) of experimental pipes smoked with *Rhus glabra* (*RGL*). The tip **(A)**, stem **(B)**, and bowl **(C)** sections were extracted with methyl *tert*-butyl ether (MTBE).

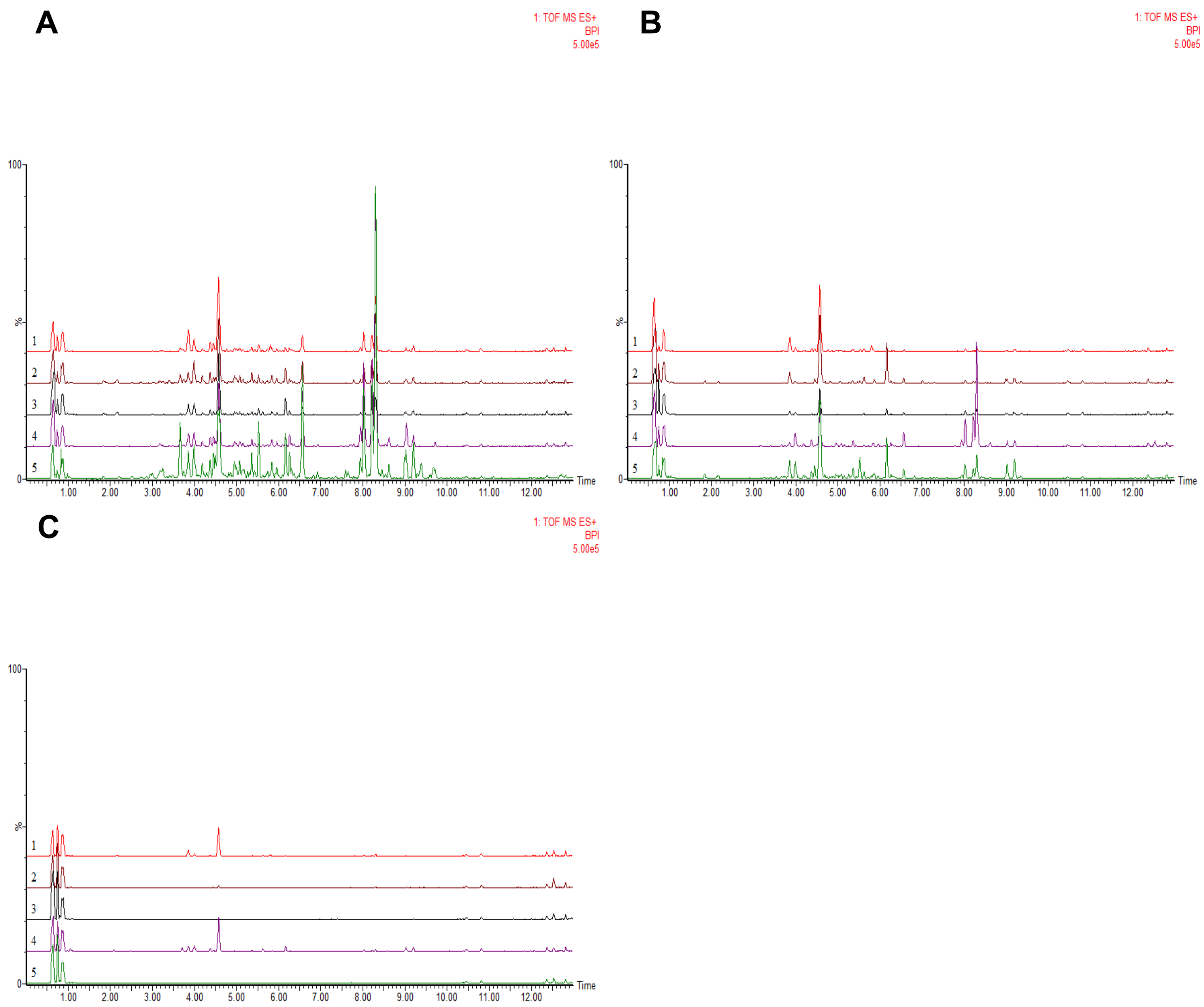

Figure S32. Each LC-MS base peak intensity (BPI) chromatogram shows five replicates (overlaid) of experimental pipes smoked with *Taxus brevifolia* (*TBR*). The tip **(A)**, stem **(B)**, and bowl **(C)** sections were extracted with 2% aqueous tartaric acid (TA).

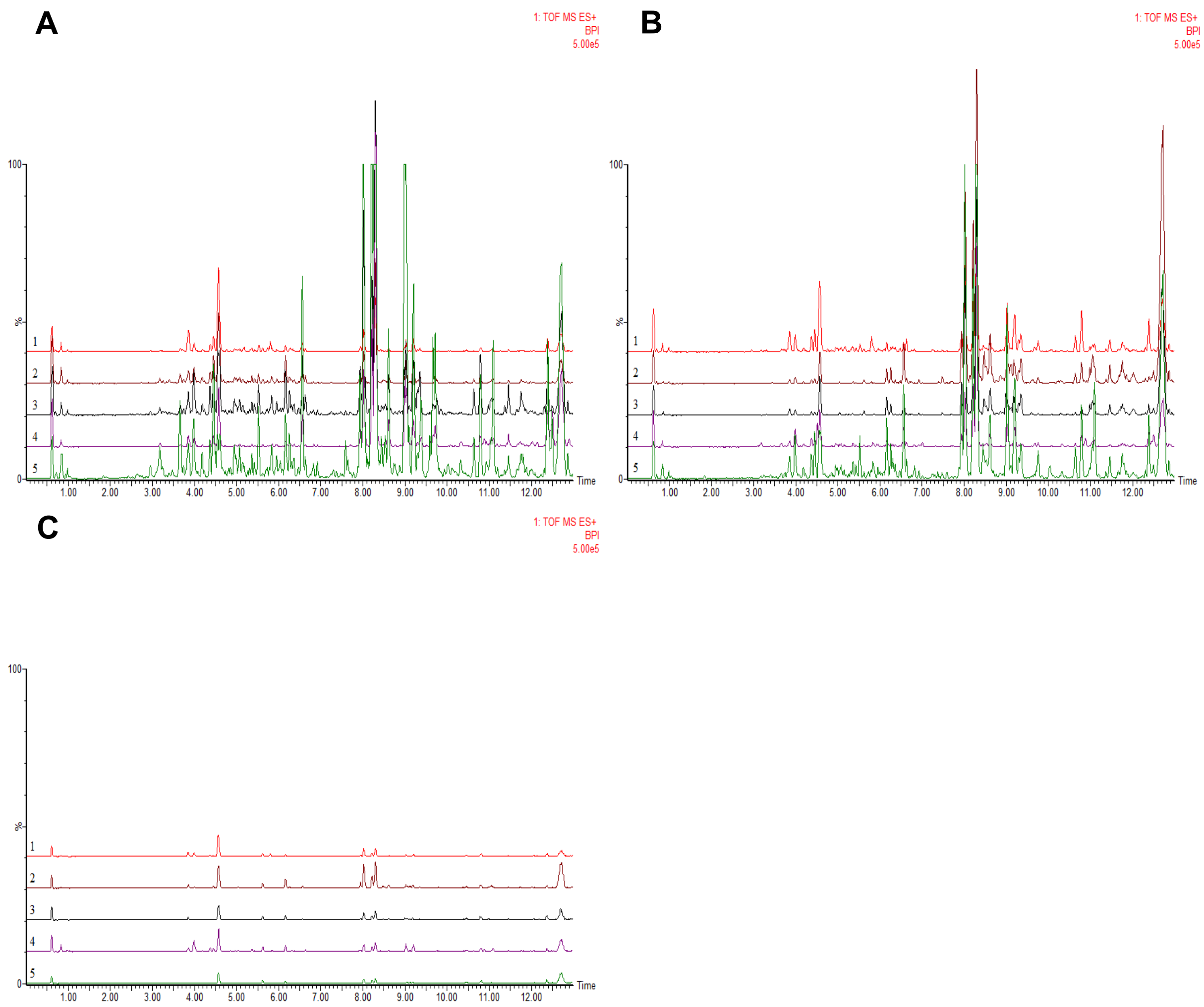

Figure S33. Each LC-MS base peak intensity (BPI) chromatogram shows five replicates (overlaid) of experimental pipes smoked with *Taxus brevifolia* (*TBR*). The tip **(A)**, stem **(B)**, and bowl **(C)** sections were extracted with acetonitrile:2-propanol:water [3:2:2] (APW).

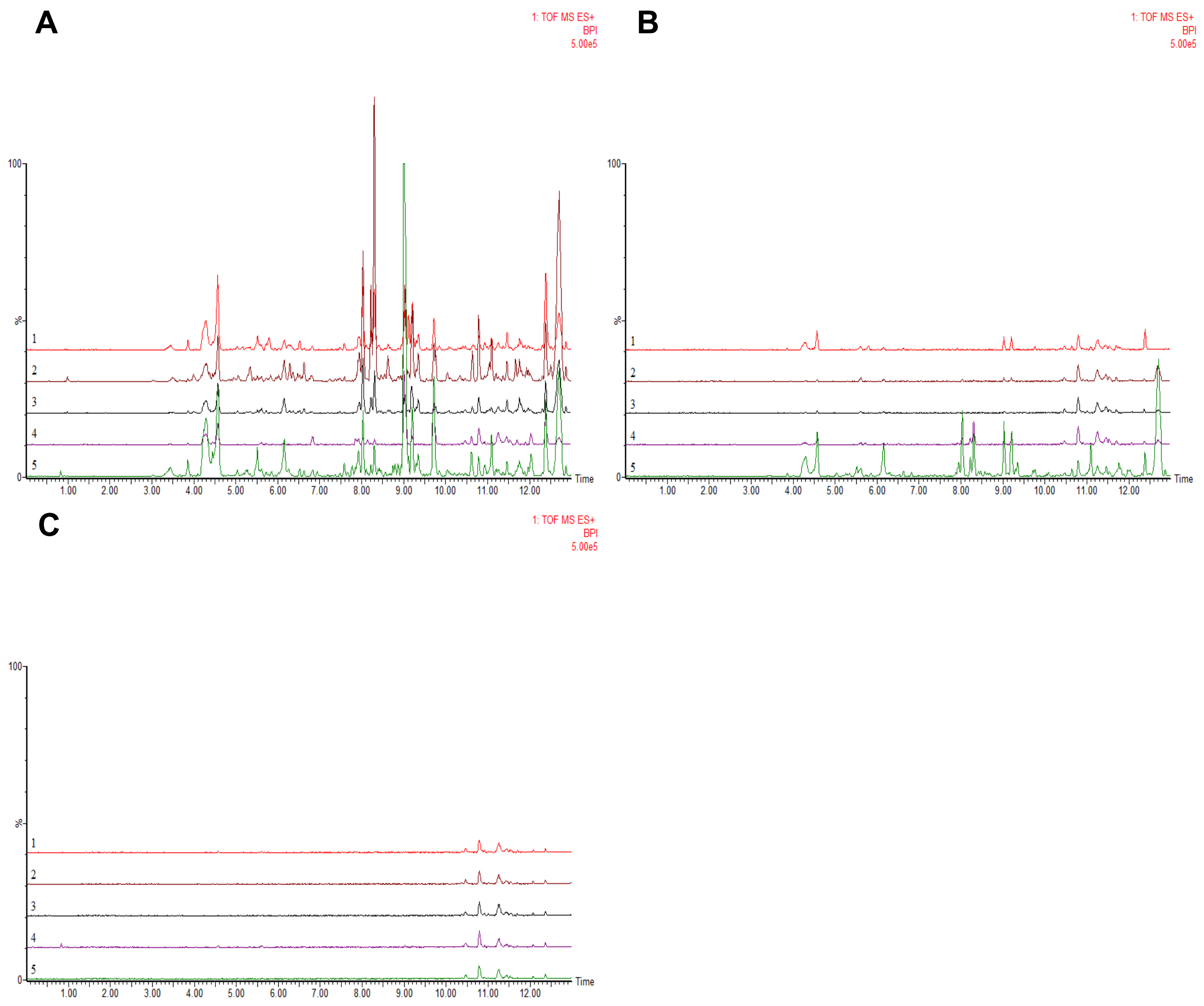

Figure S34. Each LC-MS base peak intensity (BPI) chromatogram shows five replicates (overlaid) of experimental pipes smoked with *Taxus brevifolia* (*TBR*). The tip **(A)**, stem **(B)**, and bowl **(C)** sections were extracted with methyl *tert*-butyl ether (MTBE).

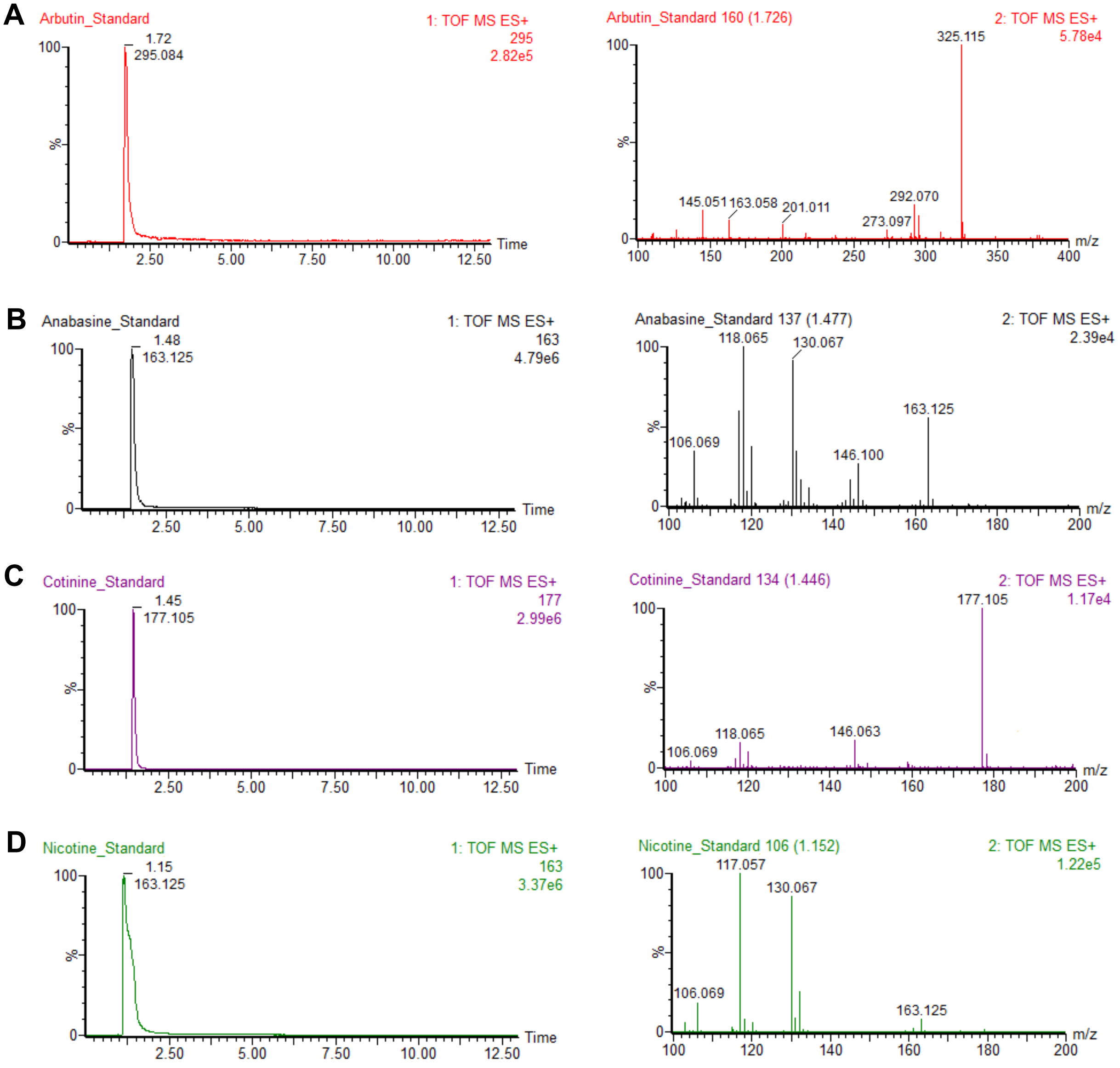
A
Arbutin_Standard
1: TOF MS ES+
295
2.82e5
1.72
295.084
Arbutin_Standard 160 (1.726)
2: TOF MS ES+
5.78e4
325.115
145.051
163.058
201.011
292.070
273.097
B
Anabasine_Standard
1: TOF MS ES+
163
4.79e6
1.48
163.125
Anabasine_Standard 137 (1.477)
2: TOF MS ES+
2.39e4
118.065
130.067
163.125
106.069
146.100
C
Cotinine_Standard
1: TOF MS ES+
177
2.99e6
1.45
177.105
Cotinine_Standard 134 (1.446)
2: TOF MS ES+
1.17e4
177.105
118.065
146.063
106.069
D
Nicotine_Standard
1: TOF MS ES+
163
3.37e6
1.15
163.125
Nicotine_Standard 106 (1.152)
2: TOF MS ES+
1.22e5
117.057
130.067
106.069
163.125
Time
m/z
%

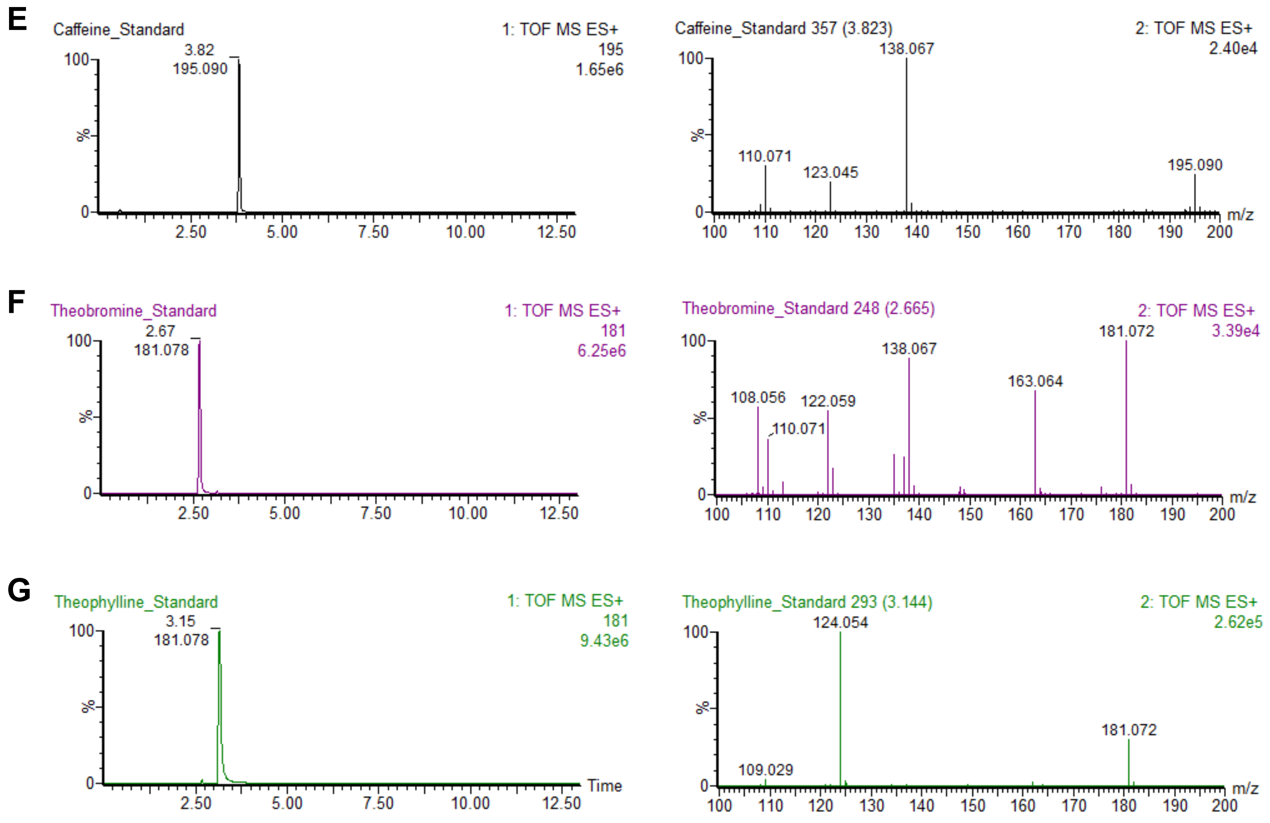

Figure S35. LC-MS chromatograms of extracted ion traces of specific precursor masses showing the retention times (left) and MS^E spectra (right) of arbutin **(A)**, anabasine **(B)**, cotinine **(C)**, nicotine **(D)**, caffeine **(E)**, theobromine **(F)**, and theophylline **(G)**.

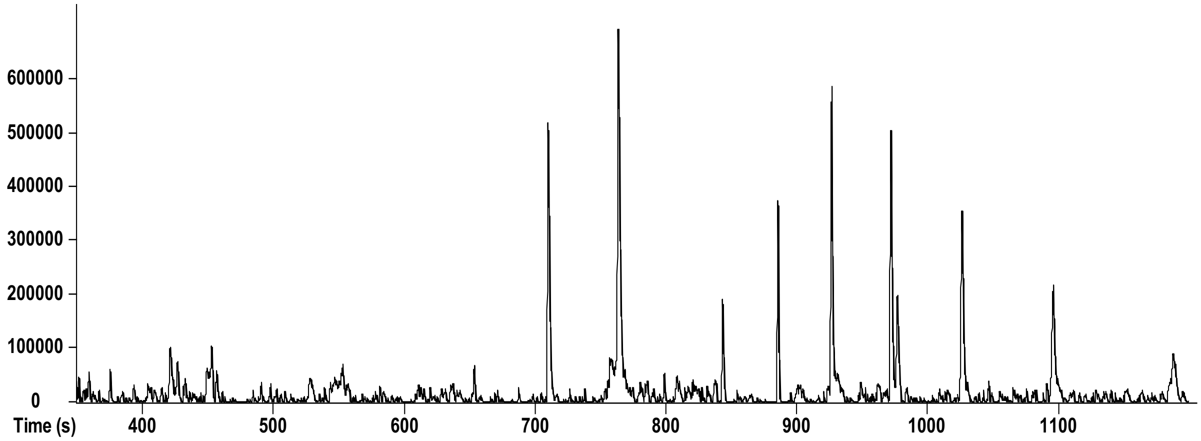

Figure S36. GC-MS analysis of the standard, theobromine. Theobromine was below the limit of detection. The peaks in the GC-MS chromatogram were determined to not be theobromine.
