# Supplementary material for: An Ancient Residue Metabolomics-Based Method to Distinguish Use of Closely Related Plant Species in Ancient Pipes
Source: Front Mol Biosci. 2020 Jun 26;7:133. doi: 10.3389/fmolb.2020.00133 (PMC7332879; doi:10.3389/fmolb.2020.00133)
Supplement: Supplementary file 2 [file Table_1.DOCX]

**Table S1.** Predicted formulas of compounds shared between the ancient pipes (i.e., PIPE_116 and PIPE_108) and plants smoked in this study. The formulas of each compound were determined using formula prediction in MZmine 2 (Pluskal et al., 2012). The parameters were set with charge: 1; ionization type: [M+H]^+^; *m/z* tolerance: 0.0 *m/z* or 3.0 ppm; element C from 0 to 50; element N from 0 to 5; element H from 0 to 100; element O from 0 to 40; and isotope pattern filter checked with isotope *m/z* tolerance: 0.0 *m/z* or 3.0 ppm; minimum absolute intensity: 1.0E4; and minimum score: 95.0%. The asterisks (*) indicate that within these parameters, the molecular formulas could not be determined. *AUV* = *A. uva-ursi*, *CSE* = *C. sericea*, *NAT* = *N. attenuata*, *NQU* = *N. quadrivalvis*, *NRU* = *N. rustica*, *NTA* = *N. tabacum*, *RGL* = *R. glabra*, and *TBR* = *T. brevifolia*.

|  | *m/z* | Retention time (min) | Predicted formula | Mass error (ppm) |
| --- | --- | --- | --- | --- |
| Common to PIPE_116 and *AUV* |  |  |  |  |
|  | 211.0799 | 8.99 | C5H12N3O6 | 0.0 |
|  | 221.0924 | 7.88 | C11H12N2O3 | 1.4 |
|  | 263.1263 | 6.50 | C13H16N3O3 | -0.6 |
|  | 277.1027 | 4.92 | * |  |
|  | 330.0558 | 0.56 | C23H7NO2 | 2.5 |
| Common to PIPE_116 and *CSE* |  |  |  |  |
|  | 193.0543 | 5.23 | CH10N3O8 | 1.1 |
|  | 264.9849 | 0.81 | C10H2NO8 | -1.7 |
|  | 291.1962 | 11.90 | C4H28N5O9 | 0.7 |
|  | 293.2121 | 11.05 | C4H30N5O9 | 1.5 |
|  | 335.2195 | 10.98 | C3H34N4O13 | -0.1 |
|  | 367.2080 | 10.93 | C2H38O19 | -0.1 |
| Common to PIPE_116 and *NQU* |  |  |  |  |
|  | 183.0921 | 3.85 | C12H10N2 | 2.2 |
|  | 211.1243 | 5.45 | C2H18N4O7 | -2.6 |
|  | 225.1382 | 6.19 | H22N3O10 | 1.7 |
|  | 227.1487 | 6.50 | C9H22O6 | -1.1 |
|  | 227.1567 | 6.37 | C3H22N4O7 | 2.4 |
|  | 261.1335 | 2.98 | C12H20O6 | 0.8 |
|  | 305.1625 | 3.41 | C17H22NO4 | 1.0 |
|  | 349.1902 | 3.78 | C7H30N3O12 | -0.1 |
| Common to PIPE_116 and *NRU* |  |  |  |  |
|  | 177.1054 | 1.05 | H18NO9 | -0.3 |
|  | 193.1708 | 5.38 | * |  |
|  | 302.8994 | 0.66 | * |  |
| Common to PIPE_116 and *NTA* |  |  |  |  |
|  | 326.3774 | 11.69 | * |  |
|  | 345.2435 | 10.75 | C8H34N5O9 | 1.6 |
| Common to PIPE_116 and *RGL* |  |  |  |  |
|  | 147.0455 | 5.01 | * |  |
|  | 149.0745 | 2.37 | * |  |
|  | 150.1252 | 3.49 | * |  |
|  | 158.1559 | 9.09 | * |  |
|  | 164.1407 | 4.65 | C8H19O3 | -0.1 |
|  | 164.1413 | 4.45 | * |  |
|  | 167.1529 | 4.45 | * |  |
|  | 169.1649 | 3.96 | C4H24O6 | 1.9 |
|  | 172.1753 | 10.13 | C3H25NO6 | -1.1 |
|  | 174.0918 | 4.86 | C11H11NO | 2.5 |
|  | 177.1054 | 3.85 | H18NO9 | -0.3 |
|  | 179.1528 | 4.32 | * |  |
|  | 181.1364 | 6.40 | H22NO9 | -2.0 |
|  | 182.1719 | 4.95 | C5H25O6 | -2.8 |
|  | 182.1719 | 5.15 | C5H25O6 | -2.8 |
|  | 185.0732 | 6.79 | * |  |
|  | 186.0930 | 3.04 | H15N3O8 | -1.2 |
|  | 187.1246 | 4.52 | H18N4O7 | -1.3 |
|  | 195.1872 | 5.36 | H26N4O7 | -1.3 |
|  | 200.2015 | 11.91 | * |  |
|  | 204.0678 | 1.36 | C14H7N2 | -2.1 |
|  | 213.1428 | 5.78 | * |  |
|  | 215.1423 | 5.21 | * |  |
|  | 217.1380 | 5.30 | C4H18N5O5 | -0.4 |
|  | 217.1949 | 11.11 | C16H24 | -0.9 |
|  | 229.1711 | 6.00 | C2H28O11 | 2.8 |
|  | 235.2060 | 11.11 | C2H28N5O7 | -0.7 |
|  | 251.1990 | 11.43 | C14H24N3O | -0.9 |
|  | 251.1990 | 11.53 | C14H24N3O | -0.9 |
|  | 261.1403 | 6.74 | C6H20N4O7 | -0.8 |
|  | 265.1789 | 11.15 | CH30NO13 | -0.4 |
|  | 289.2157 | 11.51 | C4H34NO12 | 1.0 |
|  | 291.1937 | 11.42 | CH30N4O12 | 1.3 |
| Common to PIPE_116 and *TBR* |  |  |  |  |
|  | 265.1742 | 11.25 | C9H22N5O4 | -1.1 |
|  | 291.1955 | 11.65 | C18H26O3 | 0.0 |
|  | 298.0436 | 0.60 | C11H9N2O8 | 1.4 |
|  | 325.1648 | 9.11 | C17H24O6 | 0.7 |
|  | 416.8765 | 0.67 | * |  |
| Common to PIPE_108 and *AUV* |  |  |  |  |
|  | 437.1923 | 10.84 | C8H36O19 | -0.2 |
| Common to PIPE_108 and *CSE* |  |  |  |  |
|  | 214.9094 | 0.66 | * |  |
| Common to PIPE_108 and *NRU* |  |  |  |  |
|  | 130.0638 | 1.26 | * |  |
|  | 132.0778 | 1.25 | C6H11O3 | -2.4 |
|  | 163.1155 | 0.99 | * |  |
|  | 245.1276 | 4.40 | C12H14N5O | 1.9 |
|  | 245.1341 | 4.46 | * |  |
| Common to PIPE_108 and *NTA* |  |  |  |  |
|  | 244.2008 | 7.49 | * |  |
| Common to PIPE_108 and *RGL* |  |  |  |  |
|  | 164.1407 | 4.65 | C8H19O3 | -0.1 |
|  | 229.1193 | 6.33 | * |  |
